# Antioxidant Phenylpropanoid Glycosides from *Ginkgo biloba* Fruit and Identification of a New Phenylpropanoid Glycoside, Ginkgopanoside

**DOI:** 10.3390/plants10122702

**Published:** 2021-12-08

**Authors:** Akida Alishir, Ki Hyun Kim

**Affiliations:** School of Pharmacy, Sungkyunkwan University, Suwon 16419, Korea; akida.alishir@gmail.com

**Keywords:** *Ginkgo biloba*, Ginkgoaceae, phenylpropanoid glycosides, NMR, Snatzke’s method, DPPH

## Abstract

*Ginkgo biloba* (Ginkgoaceae), well-known as the oldest living plant species and often referred to as a “living fossil,” is a famous medicinal plant that has been used in multiple countries to improve numerous illnesses, including anxiety, dementia, peripheral artery disease, and eye problems. We conducted a phytochemical exploration of *G. biloba* fruit, commonly consumed as a functional food as part of an ongoing natural product chemical research for the discovery of bioactive phytochemicals with novel structures. The natural product chemical analysis of the methanol extract of *G. biloba* fruit using column chromatography and high-performance liquid chromatography separation under the guidance of a liquid chromatography–mass spectrometry (LC/MS)-based analysis identified six phenylpropanoid glycosides (**1**–**6**), including one new compound, ginkgopanoside (**1**). The structures of the isolated compounds were elucidated by nuclear magnetic resonance spectroscopic data and LC/MS analysis, and the absolute configuration of compound **1** was established by chemical reactions followed by the application of Snatzke’s method. The 2,2-diphenyl-1-picrylhydrazyl (DPPH) scavenging activities of the isolated compounds **1**–**6** and the aglycone **1a** of **1** were evaluated, and we found that compounds **1**–**5** exhibited antioxidant activities with IC_50_ values in the range 32.75–48.20 μM, while the aglycone **1a** exhibited greater radical scavenging activity (IC_50_ = 5.23 μM) comparable to that of ascorbic acid (IC_50_ = 2.54 μM), a positive control, implying that the present of glucose may decrease the DPPH scavenging activity. These findings provide experimental information that the active phenylpropanoid glycosides could represent natural antioxidants for use in pharmaceuticals and functional foods.

## 1. Introduction

*Ginkgo biloba*, the single surviving species in the Ginkgo family (Ginkgoaceae), is native to southeastern China and is also distributed throughout Korea and Japan. Interestingly, *G. biloba* is known to be one of the oldest living plant species, often referred to as a “living fossil” [1]. *G. biloba* is considered one of the most ancient medicinal plants, and its usage in traditional medicine dates to 1505 A.D. [1]. *G. biloba* leaf extracts have been used in commercial medical products and food supplements to improve numerous illnesses, including anxiety, allergies, dementia, peripheral artery disease, and eye problems in various countries, such as China, Korea, and Japan. Several previous pharmacological studies on *G. biloba* extracts have shown that the extracts exert significant therapeutic effects on dementia by improving the cognitive performance and social functioning of patients [2]. In addition, recent studies of *G. biloba* extracts have demonstrated its beneficial activities such as neuroprotection [3] and normalization of hypertension [4]. Considering the pharmacological properties of *G. biloba* extracts, *G. biloba* has been thoroughly examined for its bioactive phytochemicals by many research groups [5,6,7,8,9], where terpene trilactones and flavonoids have been identified as the major bioactive components in *G. biloba*. Terpene trilactones, which contain ginkgolides and bilobalide as representative components, have been reported to exhibit diverse pharmacological properties [5,6]. For example, one of the representative ginkgolides, ginkgolide B, was effective in reducing the frequency of migraine attacks [7] and preventing cardiovascular disease through antioxidant activity and inhibition of platelet aggregation [8]. As another major bioactive component of *G. biloba*, flavonoids, such as myricetin and quercetin, have been shown to reduce oxidative metabolism in brain neurons [9]. Thus, diverse bioactive compounds from *G. biloba* have been attractive for natural product chemists to discover novel bioactive natural products.

As part of a continuing natural product chemical research aiming to discover bioactive novel compounds from miscellaneous natural resources [10,11,12,13], we have explored potential bioactive compounds from methanolic extracts of *G. biloba* leaves [14,15]. In our recent phytochemical investigations of these leaves, we isolated two new coumaric acid-aliphatic alcohol hybrids, namely ginkwanghols A and B, and found that both compounds promoted osteogenic differentiation by inducing the mRNA expression of the osteogenic markers *ALP* and osteopontin (*OPN*) [14]. Moreover, a new diarylpentanoid, namely ginkgobilol, and a known diarylpentanoid analog were isolated in our recent study [15], and we found that the known diarylpentanoid analog also promoted osteogenic differentiation mediated by induction of *ALP* and *OPN* gene expression, indicating its bone formation activity. As an ongoing research work for the discovery of bioactive new natural products from *G. biloba*, we moved the research material to the fruit of *G. biloba* in the present study. *G. biloba* fruit are produced by female *G. biloba* trees in the fall, and the fruit have a long history of being used as a traditional medicine for treating cough, asthma, enuresis, alcohol misuse, and pyogenic skin infections, which was first mentioned in the great herbal Pen Ts’ao Kang Mu by Li Shih-chen [16]. *G. biloba* fruit are also commonly consumed as a functional food to slow the aging process [17,18]. However, to the best of our knowledge, there are relatively few reports on bioactive compounds from the fruit of *G. biloba*, rather than the leaves. In the current study, we aimed to isolate bioactive compounds from the methanolic extracts of *G. biloba* fruit using a liquid chromatography–mass spectrometry (LC/MS)-based analysis. Six phenylpropanoid glycosides (**1**–**6**), including one new compound, ginkgopanoside (**1**), were isolated from the *n*-BuOH-soluble fraction, and we hypothesized that the isolated phenylpropanoid glycosides are the antioxidant constituents contributing to the bioactivity of *G. biloba* fruit. Herein, we describe the isolation and structural determination of isolated compounds **1**–**6** and evaluate their antioxidant activities. 

## 2. Results and Discussion

### 2.1. Isolation of Compounds **1**–**6**

**Figure 1:**
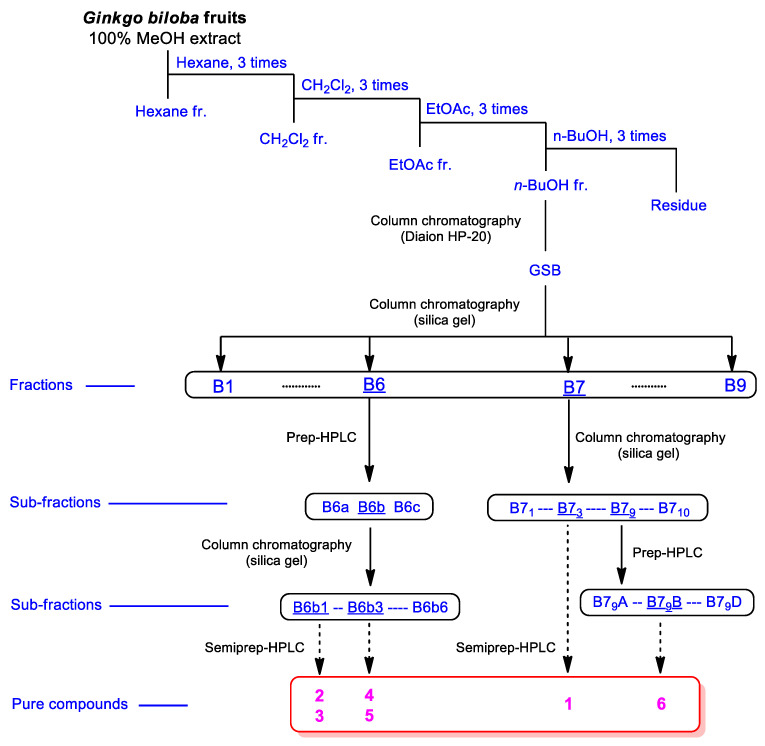
The separation scheme of compounds **1**–**6**.

**Figure 2:**
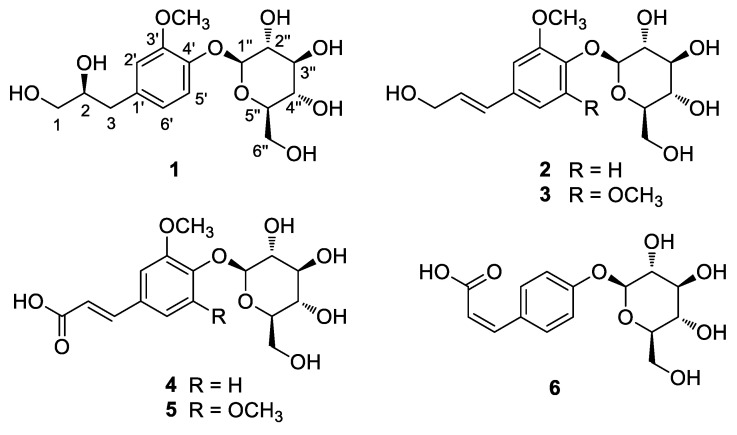
Chemical structures of compounds **1**–**6**.

Whole *G. biloba* fruit were crushed and then extracted with 100% methanol (MeOH) at room temperature to obtain the crude MeOH extract by rotary evaporation. The MeOH extract was sequentially employed in the solvent partition process with four solvents, hexane, dichloromethane, ethyl acetate, and *n*-butanol, which yielded four main solvent fractions of increasing polarity (Figure 1). LC/MS-based analysis of each fraction was carried out by reference to a house-built UV library system, which verified that the *n*-butanol-soluble fraction was rich in phenolic compounds that might have potential antioxidant activities. The exhaustive phytochemical examination of the *n*-butanol-soluble fraction by repeated column chromatography and preparative and semi-preparative high-performance liquid chromatography (HPLC; Figure 1) under the monitoring of LC/MS analysis led to the isolation of six phenylpropanoid glycosides (**1**–**6**; Figure 2). 

### 2.2. Structural Elucidation of the Isolated Compounds **1**–**6**

**Figure 3:**
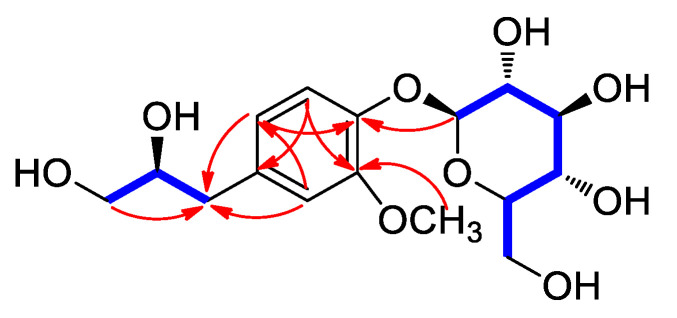
Key ^1^H-^1^H COSY (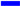) and HMBC (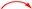) correlations for **1**.

Compound **1**, obtained as a white amorphous powder, possessed the molecular formula of C_16_H_24_O_9_ confirmed by high resolution electrospray ionisation mass spectrometry (HRESIMS) (Figure S1), which revealed a quasi-molecular ion peak at *m/z* 383.1315 [M + Na]^+^ (calculated for C_16_H_24_O_9_Na, 383.1318) in the positive-ion mode. The infrared (IR) spectrum of compound 1 showed the presence of a hydroxy group (3355 cm^−1^) and a phenyl ring (1657 and 1510 cm^−1^). The ^1^H NMR data (Table 1, Figure S2) of compound **1** showed the presence of a set of aromatic protons at *δ*_H_ 6.88 (1H, dd, *J* = 8.0, 1.5 Hz), 7.01 (1H, d, *J* = 1.5 Hz), and 7.12 (1H, d, *J* = 8.0 Hz). Furthermore, the presence of signals for one methoxy group at *δ*_H_ 3.87 (3H, s), methylene signals at *δ*_H_ 2.66 (1H, dd, *J* = 14.0, 8.5 Hz) and 2.84 (1H, dd, *J* = 14.0, 5.0 Hz), one oxygenated methine at *δ*_H_ 3.94 (1H, m), oxygenated methylene signals at *δ*_H_ 3.51 (1H, m) and 3.62 (1H, dd, *J* = 11.5, 4.0 Hz), and an indicative anomeric proton at *δ*_H_ 5.09 (1H, d, *J* = 7.5 Hz) for sugar moiety were observed. The ^13^C NMR data (Table 1), assigned by the aid of combination of heteronuclear single quantum correlation (HSQC, Figure S3) and heteronuclear multiple bond correlation (HMBC, Figure S4) spectra, showed 16 carbon resonances classified into a methoxy carbon at δ_C_ 55.6 (3′-OCH_3_), two methylene carbons at δ_C_ 64.8 (C-1) and 38.3 (C-3), an oxygenated methine carbon at δ_C_ 72.7 (C-2), six aromatic carbons at δ_C_ 116.1–148.6, and six signals assignable to the sugar moiety, including an anomeric carbon at δ_C_ 100.6 and five oxygenated carbons at δ_C_ 76.2, 75.7, 72.8, 69.2, and 60.3, which were typical of glucose [19]. The planar gross structure of compound **1** was assembled on the basis of the above considerations and the analysis of ^1^H-^1^H COSY (Figure S5) and HMBC (Figure 3). The HMBC correlations of OCH_3_/C-3′ confirmed that the methoxy group was linked to C-3′, and the HMBC correlation of H-1″/C-4′ indicated that the glucopyranose moiety was connected at C-4′. Furthermore, HMBC correlations of H-2′/C-3′, C-4′, C-6′, and C-3; H-5′/C-3′, C-4′, and C-1′; and H-6′/C-2′, C-4′, C-3 also supported the structure of compound **1** (Figure 3).

**Figure 4:**
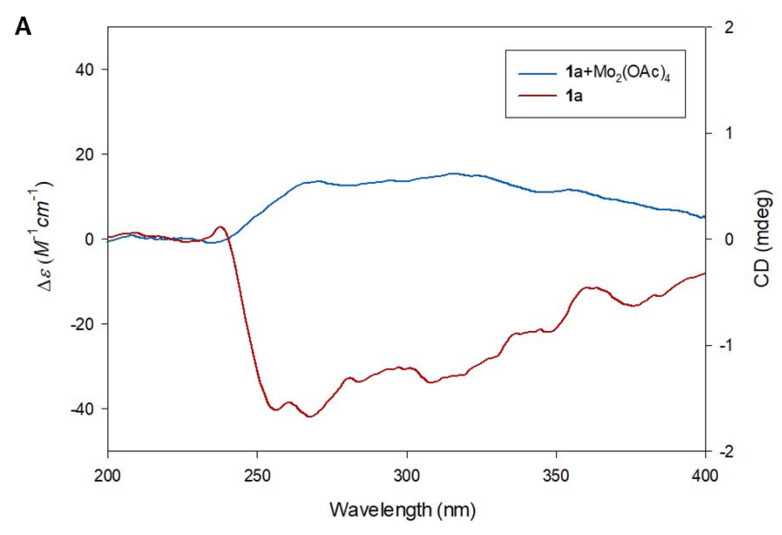

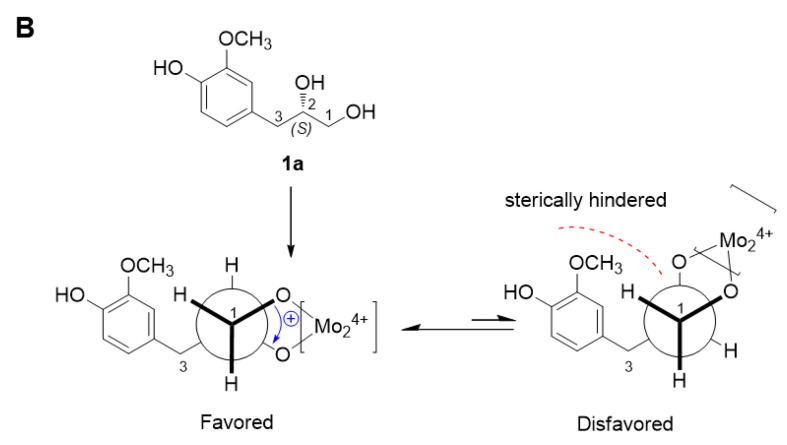
Determination of absolute configuration of C-2 in compound **1** according to Snatzke’s method. (**A**) ECD spectrum of **1a** and induced ECD spectrum of in situ formed Mo-complex of **1a** recorded in DMSO. (**B**) The favored conformations of Mo-complex of **1a**. The positive Cotton effect of the complex at 310 nm confirmed the favored conformation as being the *S*-configuration.

A literature survey revealed that the ^1^H and ^13^C NMR data of compound **1** were similar to those of piperoside [20]. However, compound 1 showed a negative optical rotation value of ${\left[\alpha \right]}_{D}^{25}$ −17.3 (*c* 0.03, MeOH), while piperoside was reported to have a positive value of ${\left[\alpha \right]}_{D}^{22}$ +186.6 (*c* 0.32, MeOH), which strongly suggests that compound **1** is an isomer of piperoside. To determine the absolute configuration of compound **1**, acid hydrolysis was carried out to obtain aglycone **1a** and the sugar moiety from **1**. The absolute configuration of sugar moiety of compound **1** was established as D-configuration by LC/MS analysis via comparison of the retention time (*t*_R_ 20.6 min) of its thiocarbamoyl-thiazolidine derivative with that (*t*_R_ 20.6 min) of the standard sample of D-glucopyranose (Figure S6) [21]. The coupling constant (*J* = 7.5 Hz) of the anomeric proton was indicative of the *β*-form glucose [22]; thereby, the sugar of compound **1** was confirmed as *β*-D-glucopyranose. Next, the absolute configuration of C-2 was determined by the application of Snatzke’s method [23,24] using the aglycone **1a** derived from acid hydrolysis of 1, because the acyclic 1,2-diol moiety is known to be difficult to assign to its absolute configuration by application of Mosher’s method and a regular electronic circular dichroism (ECD) measurement (Figure S7) [24]. After mixing aglycone **1a** and dimolybdenum tetraacetate [Mo_2_(OAc)_4_] in DMSO, a ligand–metal complex was generated as an auxiliary chromophore, for which the induced circular dichroism (ICD) spectrum was recorded and analyzed. According to Snatzke’s empirical rule [23], the absorption band at approximately 310 nm is one of the most reliably related to the absolute configuration of a 1,2-diol derivative in the [Mo_2_(OAc)_4_]-ICD spectrum. In the ICD spectrum of **1a**, the diagnostic positive Cotton effect around 310 nm corresponds to a positive dihedral angle of the O−C−C−O moiety in the favored conformation (Figure 4), which permitted the assignment of the *S*-configuration at C-2. In a previous study, aglycone **1a** was isolated from *Pimenta dioica* berries in a mixture of both *S*-form and *R*-form [25], however, it was found that the *S*-form, rather than the *R*-form, was dominant in a natural product. Accordingly, compound **1** was determined to be 2(*S*)-3-(4-*O*-*β*-D-glucopyranosyl-3-methoxyphenyl)propane-1,2-diol, and was named ginkgopanoside.

The known compounds were identified as (*E*)-coniferin (**2**) [26], syringin (**3**) [27], (*E*)-ferulic acid 4-*O-β*-D-glucoside (**4**) [28], (*E*)-sinapic acid 4-*O-β*-D-glucopyranoside (**5**) [29], and (*Z*)-4-coumaric acid 4-*O-β*-D-glucopyranoside (**6**) [30], by comparing their physical and spectroscopic data with those previously reported and LC/MS data (Figures S8–S17).

### 2.3. Evaluation of the Antioxidant Activity of Compounds **1**–**6**

Oxidative stress is characterized by an abnormally increased concentration of intracellular oxidizing species such as reactive oxygen species (ROS) [31]. Overproduction of ROS and reduced antioxidant capacity in the body are closely linked to aging, and to various diseases, including cardiovascular illness, inflammatory disorders, cancers, neurodegenerative diseases, and diabetes [32,33,34]. To date, there has been abundant evidence regarding the antioxidative effects of natural phenolic compounds, including phenylpropanoids [25,35,36,37]. Considering that all the compounds isolated from *G. biloba* fruit are phenolic compounds, the antioxidant activities of compounds **1–6**, including the aglycone **1a** of **1**, were evaluated by determining their free radical-scavenging capacities using a 2,2-diphenyl-1-picrylhydrazyl (DPPH) assay [38]. The DPPH scavenging activities are shown in Table 2. Of these compounds, all except for compound **6** exhibited weak radical-scavenging activities with IC_50_ values in the range of 32.75–48.20 μM (Table 2). Interestingly, the results showed that aglycone **1a** exhibited greater radical scavenging activity (IC_50_ = 5.23 μM) than compound **1**, comparable to the reference radical scavenger ascorbic acid (IC_50_ = 2.54 μM). This finding suggests that the presence of glucose may lessen the radical scavenging activity, which coincides with the results derived from a previous study [35].

## 3. Materials and Methods

### 3.1. General Experimental Procedure and Plant Material

Detailed information on the general experimental procedure and plant material is provided in the Supplementary Materials. 

### 3.2. Extraction and Separation/Isolation of the Compounds

Whole *G. biloba* fruit (4 kg) were crushed and then extracted with 100% MeOH (8.0 L, 5 days × 2) at room temperature, and then the filtered extract was evaporated under reduced pressure using a rotary evaporator to obtain a dried MeOH extract (425.2 g). This crude extract was suspended in distilled water (700 mL) and the solvent partitioning of the extract was conducted with hexane (700 mL), dichloromethane (CH_2_Cl_2_, 700 mL), ethyl acetate (EtOAc, 700 mL), and *n*-butanol (*n*-BuOH, 700 mL) three times. Four layers with increasing polarity, including the hexane-soluble (8.2 g), CH_2_Cl_2_-soluble (1.9 g), EtOAc-soluble (4.0 g), and *n*-BuOH-soluble layers (28.8 g) were obtained. With reference to a house-built UV library, LC/MS analysis of the four fractions from the solvent partitioning indicated the presence of the majority of phenolic compounds in the *n*-BuOH-soluble fraction. The *n*-BuOH-soluble fraction (28.8 g) was subjected to Diaion HP-20 column in 100% H_2_O to eliminate the sugar portion, and the fraction GSB (2.0 g) was obtained by elution with 100% MeOH. The fraction GSB was further subjected to silica gel column chromatography (eluted with CH_2_Cl_2_/MeOH, 20:1 to 1:1, *v*/*v*, gradient solvent system) to obtain nine fractions (B1–B9). Fraction B6 (366.4 mg) was separated by preparative reversed-phase HPLC using a gradient solvent system of MeOH-H_2_O (40–100% MeOH, flow rate of 5 mL/min) to obtain three fractions, B6a–B6c. Fraction B6b (189.1 mg) was subjected to silica gel column chromatography (eluted with CH_2_Cl_2_/MeOH, 20:1 to 1:1, *v*/*v*, gradient solvent system) to obtain six subfractions, B6b1–B6b6. Subfraction B6b1 (38.4 mg) was isolated using semi-preparative reversed-phase HPLC with 27% MeOH/H_2_O (flow rate of 2 mL/min) to obtain compounds **2** (*t*_R_ 21.5 min, 2.3 mg, 0.0005%) and **3** (*t*_R_ 27.2 min, 1.4 mg, 0.0003%). Subfraction B6b3 (46.0 mg) was isolated using semi-preparative reversed-phase HPLC with 30% MeOH/H_2_O (flow rate of 2 mL/min) to obtain compounds **4** (*t*_R_ 21.9 min, 0.8 mg, 0.0001%) and **5** (*t*_R_ 27.2 min, 1.0 mg, 0.0002%). Fraction B7 (257.0 mg) was subjected to silica gel column chromatography (eluted with CH_2_Cl_2_/MeOH, 20:1 to 1:1, *v*/*v*, gradient solvent system) to obtain ten subfractions, B7_1_–B7_10_. Subfraction B7_3_ (58.9 mg) was subjected to semi-preparative reversed-phase HPLC with 15% MeOH/H_2_O (flow rate of 2 mL/min) to isolate compound **1** (*t*_R_ 23.3 min, 5.5 mg, 0.0013%). Lastly, subfraction B7_9_ (99.7 mg) was fractionated by preparative reversed-phase HPLC using a gradient solvent system of MeOH-H_2_O (30–80% MeOH, flow rate of 5 mL/min) to obtain four subfractions, B7_9_A–B7_9_D. Subfraction B7_9_B (27.4 mg) was isolated using semi-preparative reversed-phase HPLC with 18% MeOH/H_2_O (flow rate of 2 mL/min) to yield compound **6** (*t*_R_ 83.6 min, 1.4 mg, 0.0003%). The detection of each purified compound was analyzed by an LC-MS, Agilent 1200 Series analytical system equipped with a photodiode array (PDA) detector combined with a 6130 Series ESI mass spectrometer. Each compound (0.5 mg) was dissolved in 50% aqueous MeOH (0.5 mL) and the solution was further diluted with methanol to provide a solution of 100 μg/mL. The solutions were filtered through a 0.45 mm hydrophobic PTFE filter and finally analyzed by LC/MS using a Kinetex C18 column (2.1 × 100 mm, 5 μm; Phenomenex, Torrance, CA, USA) set at 25 °C. The mobile phase consisting of formic acid in water [0.1% (*v*/*v*)] (A) and methanol (B) was delivered at a flow rate of 0.3 mL/min by applying the following programmed gradient elution: 0%-60% (B) for 10 min, 100% (B) for 1 min, 100% (B) isocratic for 5 min, and then 0% (B) isocratic for 5 min, to perform post-run reconditioning of the column. MassHunter LC/MS Software and our in-house UV library database were used for LC/MS analysis. 

#### 3.2.1. Ginkgopanoside (**1**)

White amorphous powder; ${\left[\alpha \right]}_{D}^{25}$ −17.3 (*c* 0.03, MeOH); UV (MeOH) λ_max_ (log ε) 203 (2.9), 224 (2.8), 376 (2.5) nm; IR (KBr) ν_max_ 3355, 2930, 1657, 1510, 1405, 1070 cm^−1^; ECD (MeOH) λ_max_ (Δε) 227 (−3.1), 273 (−1.4) nm; ^1^H and ^13^C NMR (850 and 212.5 MHz, respectively), see Table 1; HR-ESIMS (positive-ion mode) *m/z* 383.1315 [M + Na]^+^ (calcd for C_16_H_24_O_9_Na, 383.1318). 

#### 3.2.2. (*E*)-Coniferin (**2**)

Amorphous powder; ${\left[\alpha \right]}_{D}^{20}$ −54.8 (*c* 0.15, MeOH); UV (MeOH) λ_max_ (log ε) 215 (2.0), 225 (3.2), 260 (3.2), 297 (2.7) nm; IR (KBr) ν_max_ 3420, 2926, 1653, 1635, 1620, 1512 cm^−1^; ^1^H NMR (850 MHz, CD_3_OD): δ 7.01 (1H, d, *J* = 8.5 Hz, H-5), 6.97 (1H, d, *J* = 2.0 Hz, H-2), 6.86 (1H, dd, *J* = 8.5, 2.0 Hz, H-6), 6.46 (1H, d, *J* = 16.0 Hz, H-7), 6.18 (1H, dt, *J* = 16.0, 5.5 Hz, H-8), 4.79 (1H, d, *J* = 7.5 Hz, H-1″), 4.11 (2H, dd, *J* = 5.5, 1.5 Hz, H-9), 3.78 (3H, s, OCH_3_), 3.77 (1H, dd, *J* = 12.0, 2.0 Hz, H-6”a), 3.69 (1H, dd, *J* = 12.0, 5.0 Hz, H-6”b), 3.41–3.38 (4H, m, H-2″, 3″, 4″, 5″); ESIMS (negative-ion mode) *m/z* 387.1 [M + HCOOH − H]^−^. 

#### 3.2.3. Syringin (**3**)

Amorphous powder; ${\left[\alpha \right]}_{D}^{20}$ −15.3 (*c* 0.06, MeOH); UV (MeOH) λ_max_ (log ε) 225 (2.2), 260 (1.8) nm; IR (KBr) ν_max_ 1645, 1587, 1505 cm^−1^; ^1^H NMR (850 MHz, CD_3_OD): δ 6.66 (2H, s, H-3, 5), 6.45 (1H, d, *J* = 16.0 Hz, H-7), 6.23 (1H, dt, *J* = 16.0, 5.5 Hz, H-8), 4.78 (1H, overlapped, H-1″), 4.12 (2H, dd, *J* = 5.5, 1.5 Hz, H-9), 3.76 (6H, s, 2, 6-OCH_3_), 3.63–3.59 (2H, m, H-6”), 3.44–3.35 (4H, m, H-2”, 3”, 4”, 5”); ESIMS (negative-ion mode) *m/z* 417.1 [M + HCOOH − H]^−^. 

#### 3.2.4. (*E*)-Ferulic Acid 4-*O*-*β*-D-Glucoside (**4**)

Amorphous powder; ${\left[\alpha \right]}_{D}^{20}$ +175.5 (*c* 0.04, MeOH); UV (MeOH) λ_max_ (log ε) 225 (1.8), 285 (2.0), 315 (1.9) nm; IR (KBr) ν_max_ 3450, 1640 cm^−1^; ^1^H NMR (850 MHz, CD_3_OD): δ 7.42 (1H, d, *J* = 15.5 Hz, H-7), 7.21 (1H, d, *J* = 2.0 Hz, H-2), 7.16 (1H, dd, *J* = 8.5 Hz, H-5), 7.10 (1H, dd, *J* = 8.5, 2.0 Hz, H-6), 6.42 (1H, m, H-8), 4.95 (1H, d, *J* = 7.5 Hz, H-1′), 3.90 (3H, s, OCH_3_), 3.88 (1H, dd, *J* = 12.0, 2.0 Hz, H-6′a), 3.70 (1H, dd, *J* = 12.0, 5.5 Hz, H-6′b), 3.53–3.38 (4H, m, H-2′, 3′, 4′, 5′); ESIMS (negative-ion mode) *m/z* 355.1 [M − H]^−^. 

#### 3.2.5. (*E*)-Sinapic Acid 4-*O*-*β*-D-Glucopyranoside (**5**)

Amorphous powder; ${\left[\alpha \right]}_{D}^{25}$ –16.6 (*c* 0.05, H_2_O); UV (MeOH) λ_max_ (log ε) 203 (2.2), 234 (2.2), 300 (2.3) nm; IR (KBr) ν_max_ 3410, 1705, 1645, 1596, 1449, 1340, 1130, 1004 cm^−1^; ^1^H NMR (850 MHz, CD_3_OD): δ 7.40 (1H, d, *J* = 15.0 Hz, H-7), 6.90 (2H, s, H-2, 6), 6.47 (1H, m, H-8), 4.95 (1H, d, *J* = 7.5 Hz, H-1′), 3.88 (6H, s, 3, 5-OCH_3_), 3.78 (1H, dd, *J* = 12.0, 2.5 Hz, H-6′a), 3.66 (1H, dd, *J* = 12.0, 5.0 Hz, H-6′b), 3.53–3.41 (4H, m, H-2′, 3′, 4′, 5′); ESIMS (negative-ion mode) *m/z* 385.1 [M − H]^−^. 

#### 3.2.6. (*Z*)-4-Coumaric Acid 4-*O-β*-d-Glucopyranoside (**6**)

Amorphous powder; ${\left[\alpha \right]}_{D}^{25}$ −53.4 (*c* 0.07, H_2_O); UV (MeOH) λ_max_ (log ε) 207 (2.5), 280 (2.4) nm; IR (KBr) ν_max_ 3395, 2937, 1628, 1608, 1449, 1039 cm^−1^; ^1^H NMR (850 MHz, CD_3_OD): δ 7.57 (2H, d, *J* = 7.5 Hz, H-2, 6), 7.03 (2H, d, *J* = 8.0 Hz, H-3, 5), 6.51 (1H, d, *J* = 9.5 Hz, H-7), 5.98 (1H, d, *J* = 9.5 Hz, H-8), 4.91 (1H, d, *J* = 7.0 Hz, H-1′), 3.88 (1H, dd, *J* = 12.0, 1.5 Hz, H-6′a), 3.70 (1H, dd, *J* = 12.0, 5.5 Hz, H-6′b), 3.48–3.39 (4H, m, H-2′, 3′, 4′, 5′); ESIMS (positive-ion mode) *m/z* 349.1 [M + Na]^+^.

### 3.3. Acid Hydrolysis and Absolute Configuration Determination of the Sugar Moieties of Compound **1**

The absolute configuration of the sugar moiety was determined using a previously described method [21], with minor modifications. Compound **1** (2.0 mg) was hydrolyzed in the presence of 1 N HCl at 80 °C for 1 h, and EtOAc was used for aglycone extraction. The aqueous and EtOAc layers were neutralized by repeated evaporation using a vacuum evaporator. The dried aqueous layer was dissolved in anhydrous pyridine (0.5 mL) with the addition of L-cysteine methyl ester hydrochloride (1.0 mg). After the reaction mixture was heated at 60 °C for 1 h, *o*-tolyl isothiocyanate (50 μL) was added, and the mixture was maintained at 60 °C for 1 h. The reaction product was evaporated in a vacuum evaporator and dissolved in methanol. The dissolved reaction product was directly analyzed by LC/MS (MeOH/H_2_O, 0:10 → 8:2, gradient solvent system (0**–**30 min), 100% MeOH (31–41 min), 0% MeOH (42–52 min), and a flow rate of 0.3 mL/min), using an analytical Kinetex C_18_ 100 Å column (100 mm × 2.1 mm i.d., 5 μm). The sugar moiety in compound **1** was identified as D-glucopyranose, based on a comparison with the retention time of an authentic sample, D-glucopyranose (*t*_R_ 20.6 min) in the LC/MS analysis.

### 3.4. Absolute Configuration of the 1,2-Diol Functionalities in Compound **1**

The aglycone of **1** was obtained from EtOAc layer-derived acid hydrolysis. The aglycone **1a** was confirmed by LC/MS analysis, where the peak of **1a** with *m/z* 199.1 [M + H]^+^ was detected. According to the published procedure [23,24], 0.3 mg of the aglycone of **1** and 0.75 mg of Mo_2_(OAc)_4_ were mixed in 1.0 mL of dry DMSO with a ligand-to-metal molar ratio of approximately 1.0:1.2, and the solution was directly subjected to ECD measurements. The first circular dichroism (CD) spectrum was recorded immediately after mixing, and its time evolution was monitored until it was stationary (approximately 30 min after mixing). The inherent CD was subtracted. The observed signs of the diagnostic band at approximately 310 nm in the induced CD spectra were correlated with the absolute configuration of the 1,2-diol moiety.

### 3.5. DPPH Radical-Scavenging Assay

The antioxidant activities of compounds **1**–**6** and aglycone **1a** were evaluated by their free radical-scavenging capacities using the DPPH assay [38]. In microwells, 100 μL of an aqueous solution of completely dissolved sample (control: 100 μL of distilled water) were added to an ethanolic solution of DPPH (100 μL, 60 μM), which was incubated for 15 min at room temperature in the dark. The final concentrations of the tested samples in the assayed solutions were 5, 10, 25 and 50 μM. Ascorbic acid was used as the standard for comparison. The ability to scavenge DPPH radicals was calculated in terms of percentage inhibition according to the following equation: % inhibition = [(A_0_ − A_1_)/A_0_ × 100], where A_0_ is the absorbance of the control (without sample) and A_1_ is the absorbance in the presence of the sample. 

## 4. Conclusions

In this study, we isolated and characterized six phenylpropanoid glycosides (**1**–**6**), including one new compound, ginkgopanoside (**1**), in a polar fraction of the methanolic extracts of *G. biloba* fruit via an LC/MS-based analysis. The structure of ginkgopanoside was established by NMR spectroscopic methods and HR-ESIMS, and its absolute configuration was confirmed by chemical reactions followed by the application of Snatzke’s method. We revealed that compounds **1**–**5** showed antioxidant activities with IC_50_ values in the range of 32.75–48.20 μM, while the aglycone **1a** exhibited greater radical scavenging activity (IC_50_ = 5.23 μM) comparable to that of ascorbic acid (IC_50_ = 2.54 μM), implying that the present of glucose may lessen the DPPH scavenging activity. The structure–activity relationship information will facilitate future synthetic and pharmacological studies for developing novel antioxidant drugs. The present study suggests future possibility that active phenylpropanoid glycosides can be potential sources of natural antioxidants for use in pharmaceuticals and functional foods. 

## Figures and Tables

**Table 1:** ^1^H (850 MHz) and ^13^C NMR (212.5 MHz) data of compound **1** in D_2_O (*δ* in ppm) ^a^.

Position	1	
	*δ*_H_ (*J* in Hz)	*δ*_C_
1	3.51, m; 3.62, dd (11.5, 4.0)	64.8, CH_2_
2	3.94, m	72.7, CH
3	2.66, dd (14.0, 8.5); 2.84, dd (14.0, 5.0)	38.3, CH_2_
1′		134.1, C
2′	7.01, d (1.5)	113.8, CH
3′		148.6, C
4′		143.8, C
5′	7.12, d (8.0)	116.1, CH
6′	6.88, dd (8.0, 1.5)	122.1, CH
3′-OCH_3_	3.87, s	55.6, CH_3_
1′′	5.09, d (7.5)	100.6, CH
2′′	3.60, m	72.8, CH
3′′	3.51, m	69.2, CH
4′′	3.57, m	76.2, CH
5′′	3.58, m	75.7, CH
6′′	3.75, dd (12.5, 5.5); 3.90, dd (12.5, 2.0)	60.3, CH_2_

^a^*J* values are in parentheses and shown in Hz; ^13^C NMR assignments are based on HSQC and HMBC experiments.

**Table 2:** Antioxidant activities of compounds **1**–**6** and **1a** in a DPPH radical-scavenging assay.

Compound	IC_50_ (μM) ^a^	Compound	IC_50_ (μM) ^a^
1	32.75 ± 2.32	4	37.85 ± 5.10
1a	5.23 ± 0.78	5	45.83 ± 1.90
2	36.51 ± 1.42	6	>50
3	48.20 ± 3.08	Ascorbic acid ^b^	2.54 ± 0.21

^a^ IC_50_ value of each compound is presented as mean ± SEM of triplicate determination. ^b^ Ascorbic acid as a positive control.

## Supplementary Materials

The following are available online at https://www.mdpi.com/article/10.3390/plants10122702/s1, Figure S1: High-resolution electrospray ionization mass spectroscopy (HRESIMS) data of 1; Figure S2: 1H nuclear magnetic resonance (NMR) spectrum of 1 (D2O, 850 MHz); Figure S3: heteronuclear single quantum coherence (HSQC) spectrum of 1 (D2O); Figure S4: The heteronuclear multiple bond correlation (HMBC) spectrum of 1 (D2O); Figure S5: The 1H-1H correlation spectroscopy (COSY) spectrum of 1 (D2O); Figure S6: Retention time of reaction products of thiocarbamoyl-thiazolidine derivatives of D-glucopyranose standard and glucopyranose from 1; Figure S7: Circular dichroism spectrum of 1; Figure S8: The ESIMS data of 2; Figure S9: The 1H NMR spectrum of 2 (CD3OD, 850 MHz); Figure S10: The ESIMS data of 3; Figure S11: The 1H NMR spectrum of 3 (CD3OD, 850 MHz); Figure S12: The ESIMS data of 4; Figure S13: The 1H NMR spectrum of 4 (CD3OD, 850 MHz); Figure S14: The ESIMS data of 5; Figure S15: The 1H NMR spectrum of 5 (CD3OD, 850 MHz); Figure S16: The ESIMS data of 6; Figure S17: The ^1^H NMR spectrum of **6** (CD_3_OD, 850 MHz); General experimental procedure.

## References

[1] Jacobs B.P., Browner W.S. (2000). *Ginkgo biloba*: A living fossil. Am. J. Med..

[2] Renzo G.D. (2000). *Ginkgo biloba* and the central nervous system. Fitoterapia.

[3] Shah Z.A., Nada S.E., Doré S. (2011). Heme oxygenase 1, beneficial role in permanent ischemic stroke and in Gingko biloba (EGb 761) neuroprotection. Neuroscience.

[4] Mansour S.M., Bahgat A.K., El-Khatib A.S., Khayyal M.T. (2011). *Ginkgo biloba* extract (EGb 761) normalizes hypertension in 2K, 1C hypertensive rats: Role of antioxidant mechanisms, ACE inhibiting activity and improvement of endothelial dysfunction. Phytomedicine.

[5] Sabater-Jara A.B., Souliman-Youssef S., Novo-Uzal E., Almagro L., Belchí-Navarro S., Pedreño M.A. (2013). Biotechnological approaches to enhance the biosynthesis of ginkgolides and bilobalide in *Ginkgo biloba*. Phytochem Rev..

[6] Goto H., Usuki T. (2012). ^1^H-NMR Analysis of Terpene Trilactones (TTLs) in *Ginkgo biloba*: Green Female Leaves Contain the Most TTLs. Phytochem. Anal..

[7] Usai S., Grazzi L., Bussone G. (2011). Gingkolide B as migraine preventive treatment in young age: Results at 1-year follow-up. Neurol Sci..

[8] Mahady G.B. (2002). *Ginkgo Biloba* for the Prevention and Treatment of Cardiovascular Disease: A Review of the Literature. J. Cardiovasc. Nurs..

[9] Oyama Y., Fuchs P.A., Katayama N., Noda K. (1994). Myricetin and quercetin, the flavonoid constituents of *Ginkgo biloba* extract, greatly reduce oxidative metabolism in both resting and Ca^2+^-loaded brain neurons. Brain Res..

[10] Lee S.R., Kang H.S., Yoo M.J., Yi S.A., Beemelmanns C., Lee J.C., Kim K.H. (2020). Anti-adipogenic Pregnane Steroid from a Hydractinia-associated Fungus, *Cladosporium sphaerospermum* SW67. Nat. Prod. Sci..

[11] Lee S., Ryoo R., Choi J.H., Kim J.H., Kim S.H., Kim K.H. (2020). Trichothecene and tremulane sesquiterpenes from a hallucinogenic mushroom *Gymnopilus junonius* and their cytotoxicity. Arch. Pharm. Res..

[12] Ha J.W., Kim J., Kim H., Jang W., Kim K.H. (2020). Mushrooms: An Important Source of Natural Bioactive Compounds. Nat. Prod. Sci..

[13] Yu J.S., Park M., Pang C., Rashan L., Jung W.H., Kim K.H. (2020). Antifungal phenols from *Woodfordia uniflora* Collected in Oman. J. Nat. Prod..

[14] Lee K.H., Kim J.K., Yu J.S., Jeong S.Y., Choi J.H., Kim J.-C., Ko Y.-J., Kim S.-H., Kim K.H. (2021). Ginkwanghols A and B, osteogenic coumaric acid-aliphatic alcohol hybrids from the leaves of *Ginkgo biloba*. Arch. Pharm. Res..

[15] Lee K.H., Yu J.S., Choi J.H., Kim S.-H., Ko Y.-J., Pang C., Kim K.H. (2020). Ginkgobilol, a new diarylpentanoid and an osteogenic diarylpentanoid analog from *Ginkgo biloba* leaves. Bioorganic Med. Chem. Lett..

[16] Li H.L. (1956). A horticultural and botanical history of Ginkgo. Morris Arbor. bull..

[17] Pan J.X., Zhang H.Y., Yang X.B. (1995). Biflavones from the testa of *Ginkgo biloba* L. J. Plant Resour. Environ..

[18] Lou F., Wang G., Guo Y. (1998). Chemical study on the exopleura of *Ginkgo biloba* L. J. China Pharm. Univ..

[19] Harinantenaina L.R.R., Kasai R., Yamasaki K. (2002). *Ent*-kaurane Diterpenoid Glycosides from the Leaves of *Cussonia racemosa*, a Malagasy Endemic Plant *Chem*. Pharm. Bull..

[20] Luyen B.T.T., Tai B.H., Thao N.P., Yang S.Y., Cuong N.M., Kwon Y.I., Jang H.D., Kim Y.H. (2014). A new phenylpropanoid and an alkylglycoside from *Piper retrofractum* leaves with their antioxidant and α-glucosidase inhibitory activity. Bioorg. Med. Chem. Lett..

[21] Tanaka T., Nakashima T., Ueda T., Tomii K., Kouno I. (2007). Facile Discrimination of Aldose Enantiomers by Reversed-Phase HPLC. Chem. Pharm. Bull..

[22] Coxon B. (1983). Two-Dimensional *J*-Resolved Proton Nuclear Magnetic Resonance Spectrometry of Hydroxyl-Coupled *α*- and *β*-D-Glucose. Anal. Chem..

[23] Di Bari L., Pescitelli G., Pratelli C., Pini D., Salvadori P. (2001). Determination of Absolute Configuration of Acyclic 1,2-Diols with Mo_2_(OAc)_4_. 1. Snatzke’s Method Revisited. J. Org. Chem..

[24] Pan L., Acuña UM., Li J., Jena N., Ninh T.N., Pannell C.M., Chai H., Fuchs J.R. (2013). Carcache de Blanco, E.J., Soejarto, D.D., Kinghorn, A.D. Bioactive Flavaglines and Other Constituents Isolated from *Aglaia perviridis*. J. Nat. Prod..

[25] Kikuzaki H., Hara S., Kawai Y., Nakatani N. (1999). Antioxidative phenylpropanoids from berries of *Pimenta dioica*. Phytochemistry..

[26] Chen Y.-G., Yu L.-L., Huang R., Lv Y.-P., Gui S.-H. (2005). 11-Methoxyviburtinal, a new iridoid from *Valeriana jatamansi*. Arch. Pharm. Res..

[27] Wu L., Shen Y., Zheng J., Wu B., Zheng L. (1999). The NMR study of syringin. Chinese J. Magn. Reson..

[28] Kurkin V.A., Lamrini M., Klochkov S.G. (2008). Lavandoside from *Lavandula spica* flowers. Chem. Nat. Compd..

[29] Wolfram K., Schmidt J., Wray V., Milkowski C., Schliemann W., Strack D. (2010). Profiling of phenylpropanoids in transgenic low-sinapine oilseed rape (*Brassica napus*). Phytochemistry.

[30] Foo L.Y., Lu Y., Molan A.L., Woodfield D.R., McNabb W.C. (2000). The phenols and prodelphinidins of white clover flowers. Phytochemistry.

[31] Collins C.A., Fry F.H., Holme A.L., Yiakouvaki A., Al-Qenaei A., Pourzand C., Jacob C. (2005). Towards multifunctional antioxidants: Synthesis, electrochemistry, *in vitro* and cell culture evaluation of compounds with ligand/catalytic properties. Org. Biomol. Chem..

[32] Finkel T., Holbrook N.J. (2000). Oxidants, oxidative stress and the biology of ageing. Nature.

[33] Choudhry Q.N., Kim J.H., Cho H.T., Heo W., Lee J.J., Lee J.H., Kim Y.J. (2019). Ameliorative effect of black ginseng extract against oxidative stress-induced cellular damages in mouse hepatocytes. J. Ginseng Res..

[34] Lee H., Shahbaz H.M., Ha N., Kim J.U., Lee S.J., Park J. (2020). Development of ginseng powder using high hydrostatic pressure treatment combined with UV-TiO2 photocatalysis. J. Ginseng Res..

[35] Wojdyło A., Nowicka P., Grimalt M., Legua P., Almansa M.S., Amorós A., Carbonell-Barrachina Á.A., Hernández F. (2019). Polyphenol Compounds and Biological Activity of Caper (*Capparis spinosa* L.) Flowers Buds. Plants.

[36] Iannuzzi A.M., Giacomelli C., De Leo M., Pietrobono D., Camangi F., De Tommasi N., Martini C., Trincavelli M.L., Braca A. (2020). Antioxidant Activity of Compounds Isolated from *Elaeagnus umbellata* Promotes Human Gingival Fibroblast Well-Being. J. Nat. Prod..

[37] Jang J.Y., Ahn J.H., Jo Y.H., Hwang B.Y., Lee M.K. (2019). Antioxidant Activity and Phenolic Content of Different Parts of Lotus and Optimization of Extraction Condition using Response Surface Methodology. Nat. Prod. Sci..

[38] Pan H.Y., Qu Y., Zhang J.K., Kang T.G., Dou D.Q. (2013). Antioxidant activity of ginseng cultivated under mountainous forest with different growing years. J. Ginseng Res..

