# Supplementary material for: Antioxidant Phenylpropanoid Glycosides from Ginkgo biloba Fruit and Identification of a New Phenylpropanoid Glycoside, Ginkgopanoside
Source: Plants (Basel). 2021 Dec 8;10(12):2702. doi: 10.3390/plants10122702 (PMC8708797; doi:10.3390/plants10122702)
Supplement: Supplementary file 1 [file plants-10-02702-s001.zip › plants-1500844-supplementary.pdf]

Supplementary data

Antioxidant Phenylpropanoid Glycosides from *Ginkgo biloba* Fruit and Identification of a New Phenylpropanoid Glycoside, Ginkgopanoside

Akida Alishir, and Ki Hyun Kim*

School of Pharmacy, Sungkyunkwan University, Suwon 16419, Korea; akida.alishir@gmail.com (A.A.)

* Corresponding author: khkim83@skku.edu; Tel.: +82-31-290-7700

Supporting Information Contents:

Figure S1. The HRESIMS data of **1**……S3
Figure S2. The ^{1}H NMR spectrum of **1** (D_2O, 850 MHz)……S4
Figure S3. The HSQC spectrum of **1** (D_2O)……S5
Figure S4. The HMBC spectrum of **1** (D_2O)……S6
Figure S5. The ^{1}H-^{1}H COSY spectrum of **1** (D_2O)……S7
Figure S6. Retention time of reaction products of thiocarbamoyl-thiazolidine derivatives of D-glucopyranose standard and glucopyranose from **1** ……S8
Figure S7. The circular dichroism spectrum of **1**……S9
Figure S8. The ESIMS data of **2**……S10
Figure S9. The ^{1}H NMR spectrum of **2** (CD_3OD, 850 MHz)……S11
Figure S10. The ESIMS data of **3**……S12
Figure S11. The ^{1}H NMR spectrum of **3** (CD_3OD, 850 MHz)……S13
Figure S12. The ESIMS data of **4**……S14
Figure S13. The ^{1}H NMR spectrum of **4** (CD_3OD, 850 MHz)……S15
Figure S14. The ESIMS data of **5**……S16
Figure S15. The ^{1}H NMR spectrum of **5** (CD_3OD, 850 MHz)……S17
Figure S16. The ESIMS data of **6**……S18
Figure S17. The ^{1}H NMR spectrum of **6** (CD_3OD, 850 MHz)……S19
General experimental procedure……S20

Figure S1. The HRESIMS data of **1**

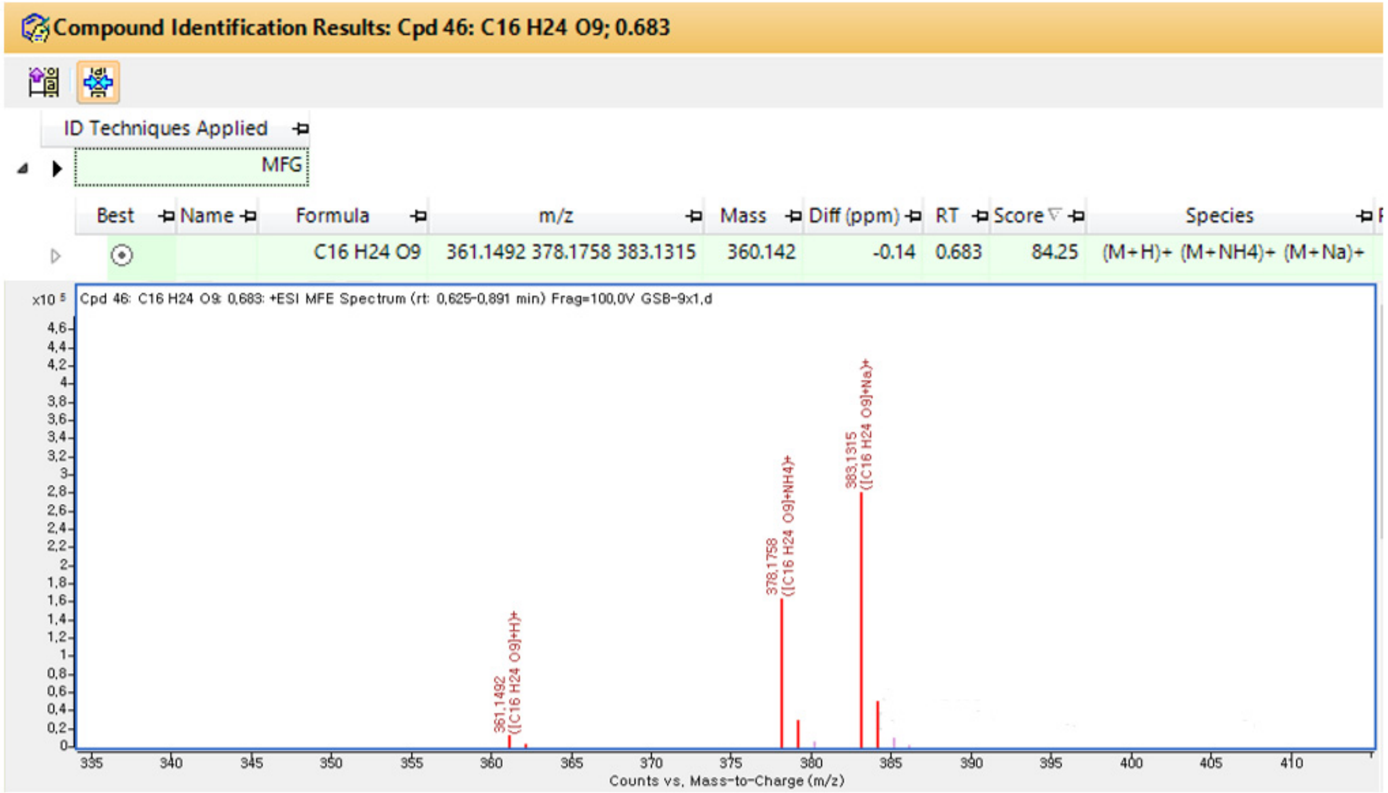

Figure S2. The ^{1}H NMR spectrum of **1** (D_2O, 850 MHz)

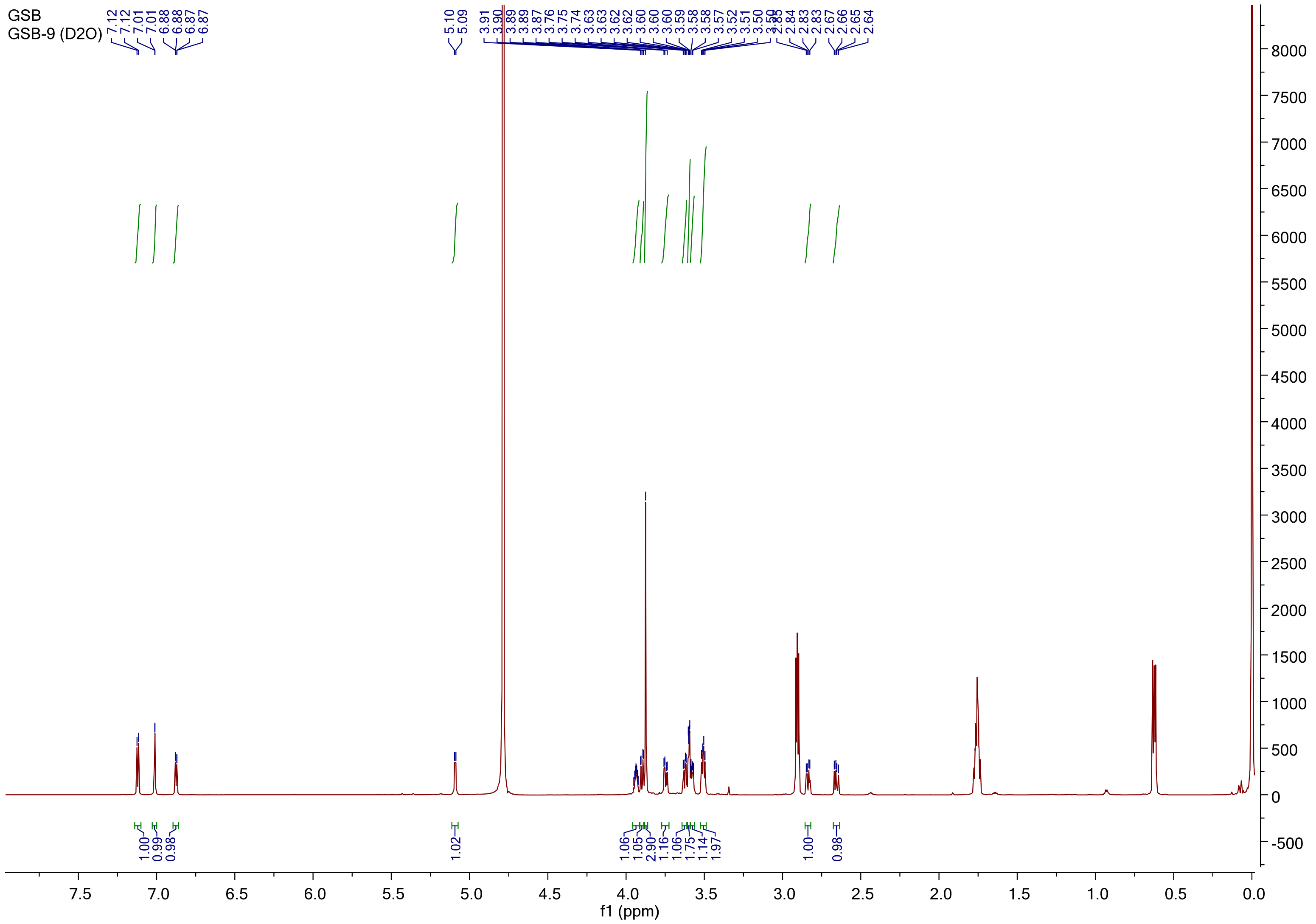

Figure S3. The HSQC spectrum of **1** (D_2O)

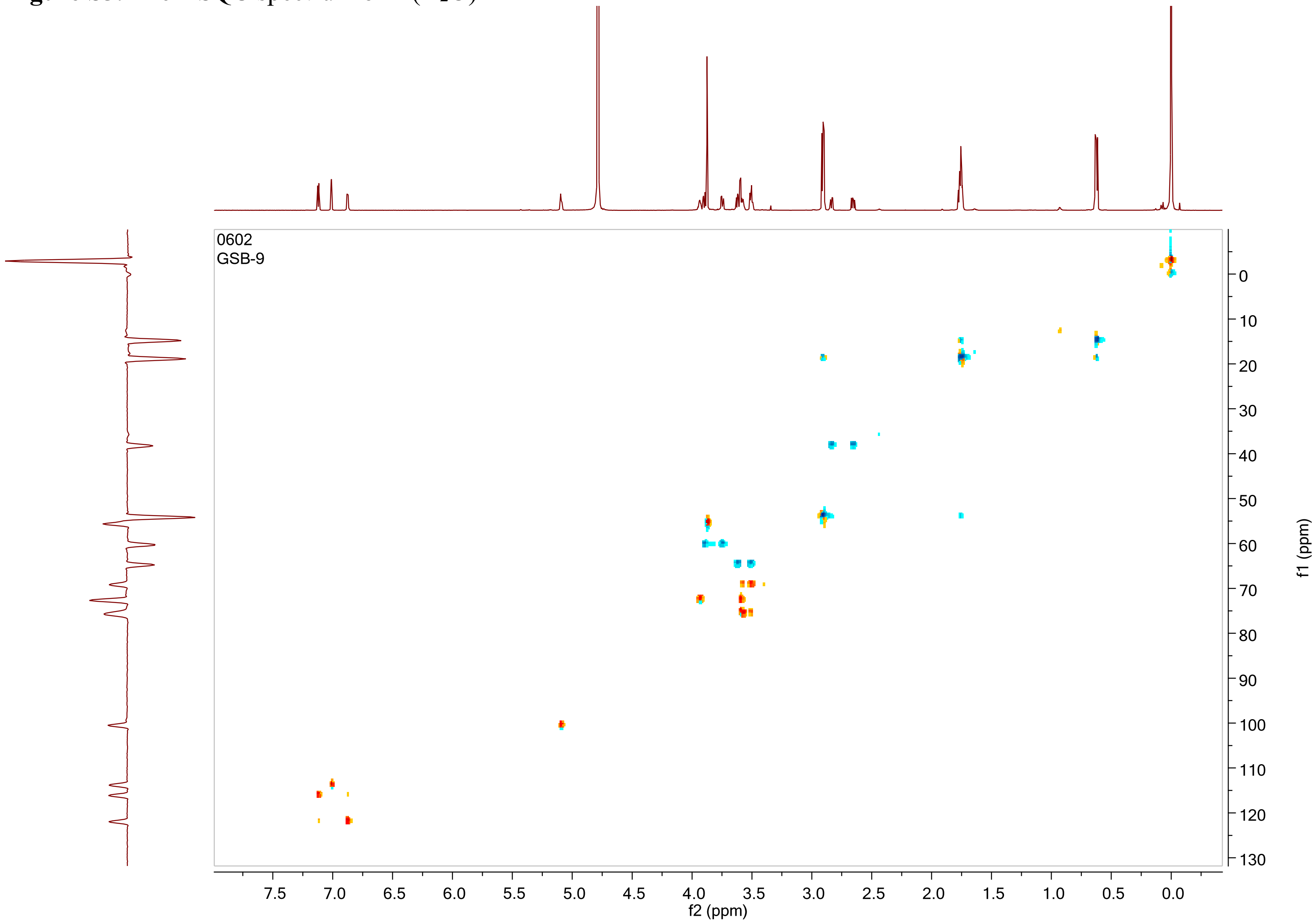

Figure S4. The HMBC spectrum of **1** (D_2O)

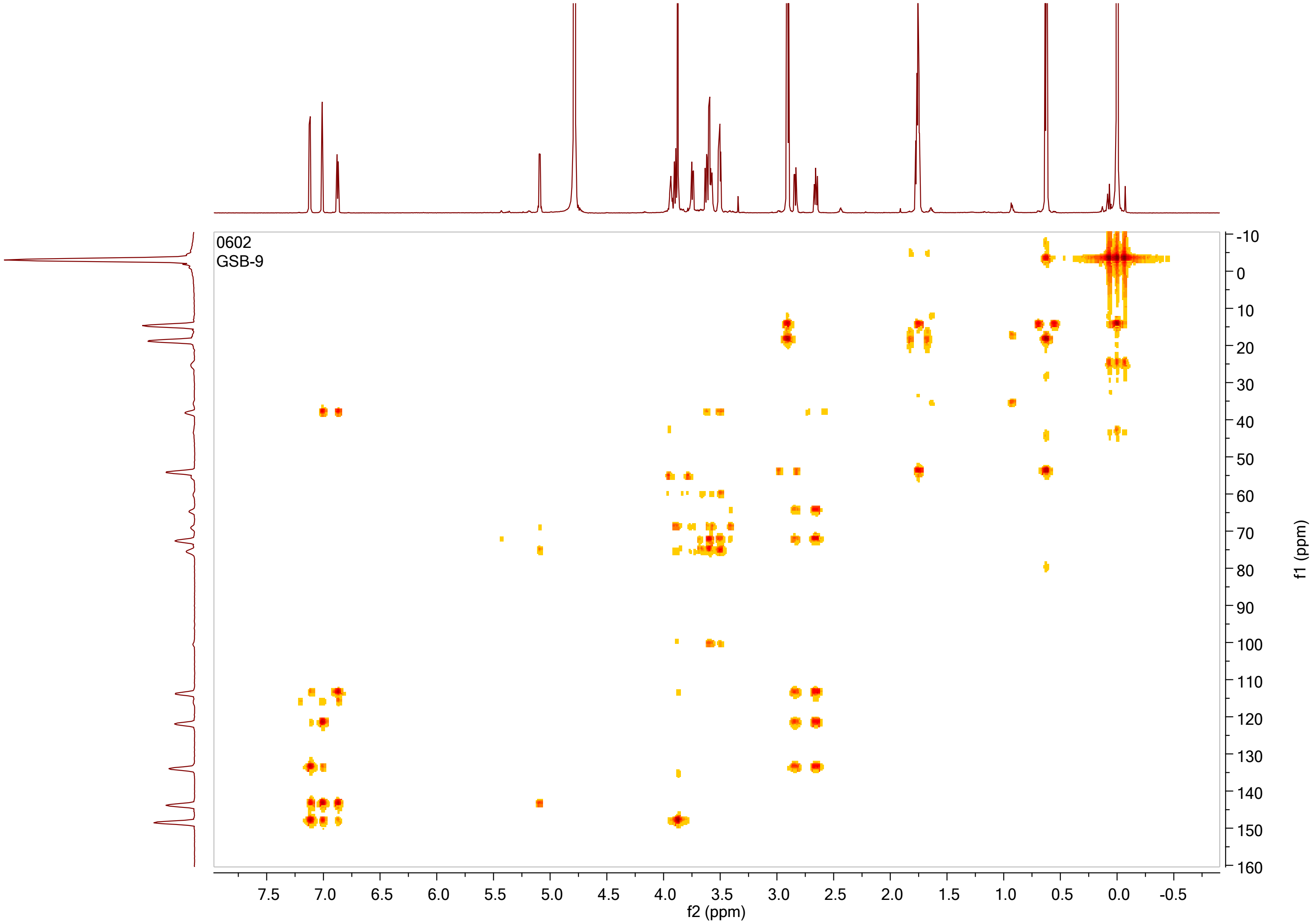

Figure S5. The ^{1}H-^{1}H COSY spectrum of **1** (D_2O)

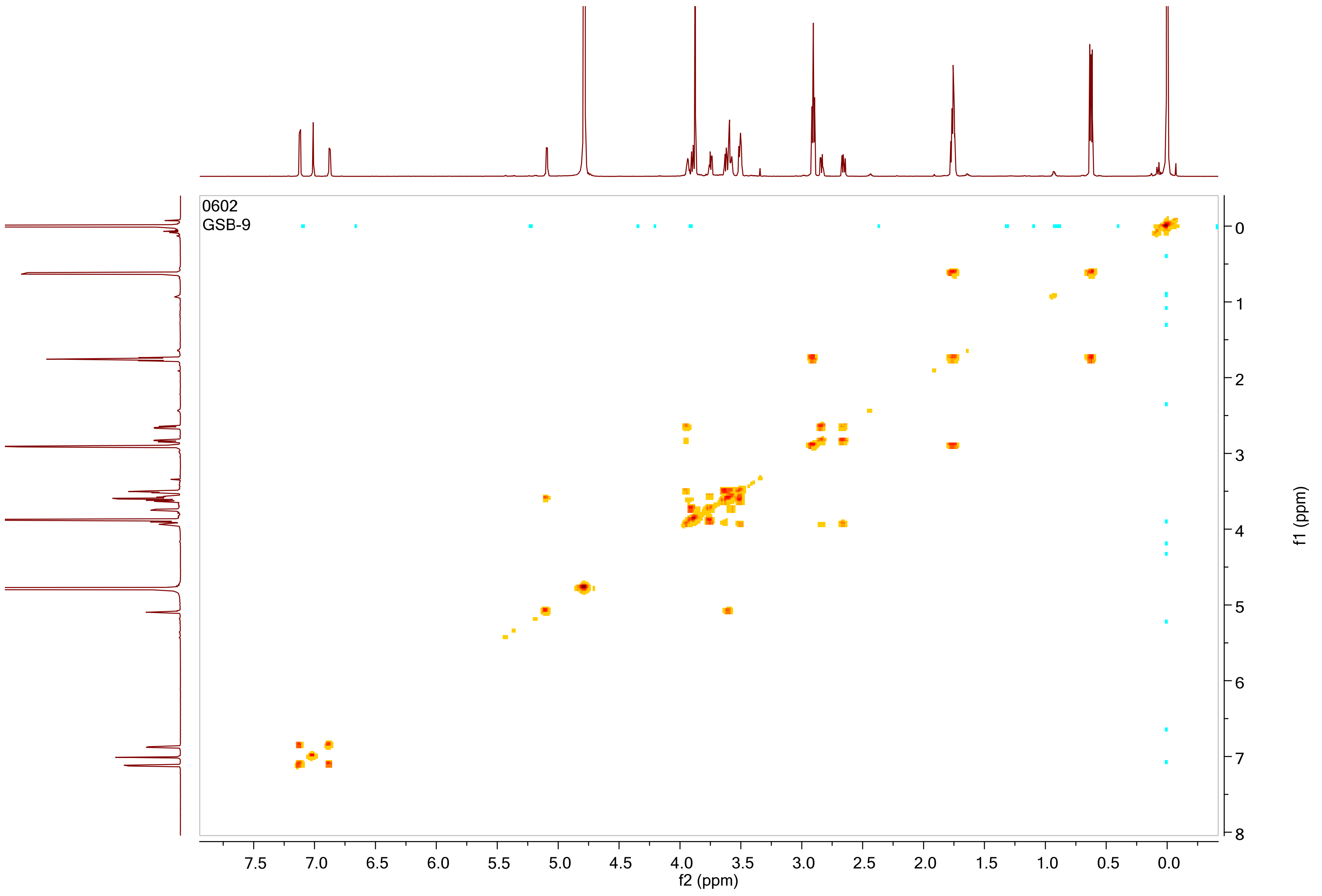

Figure S6. Retention time of reaction products of thiocarbamoyl-thiazolidine derivatives of D-glucopyranose standard and glucopyranose from compound **1**

Positive

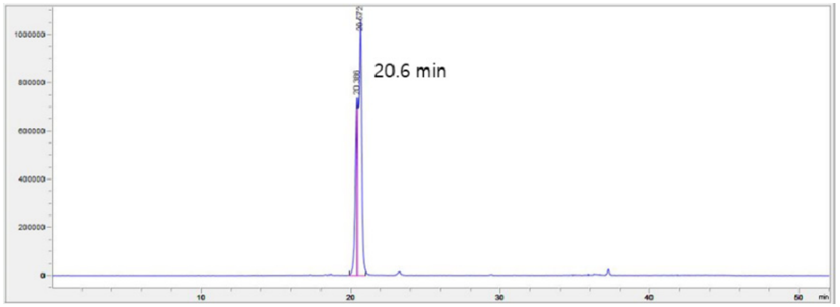

Reaction product of D-glucopyrancose standard

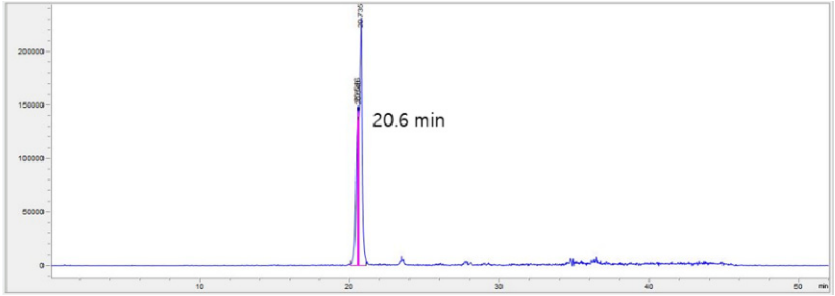

Reaction product of glucopyrancose from compound 1

Negative

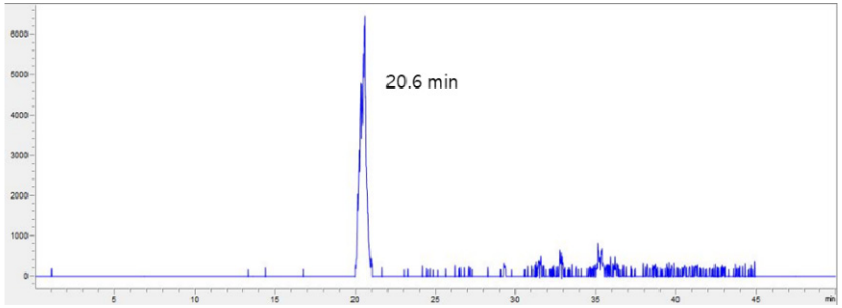

Reaction product of D-glucopyrancose standard

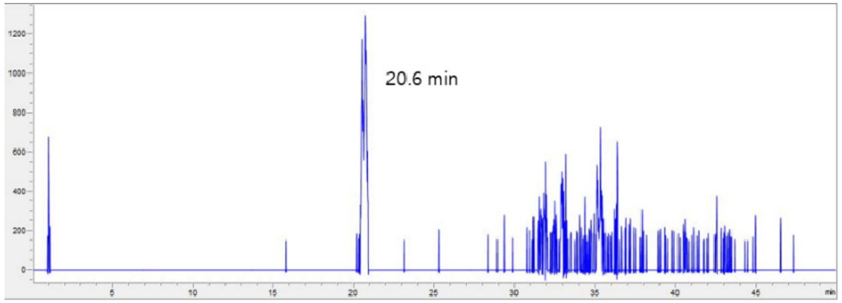

Reaction product of glucopyrancose from compound **1**

Figure S7. The circular dichroism spectrum of compound **1**

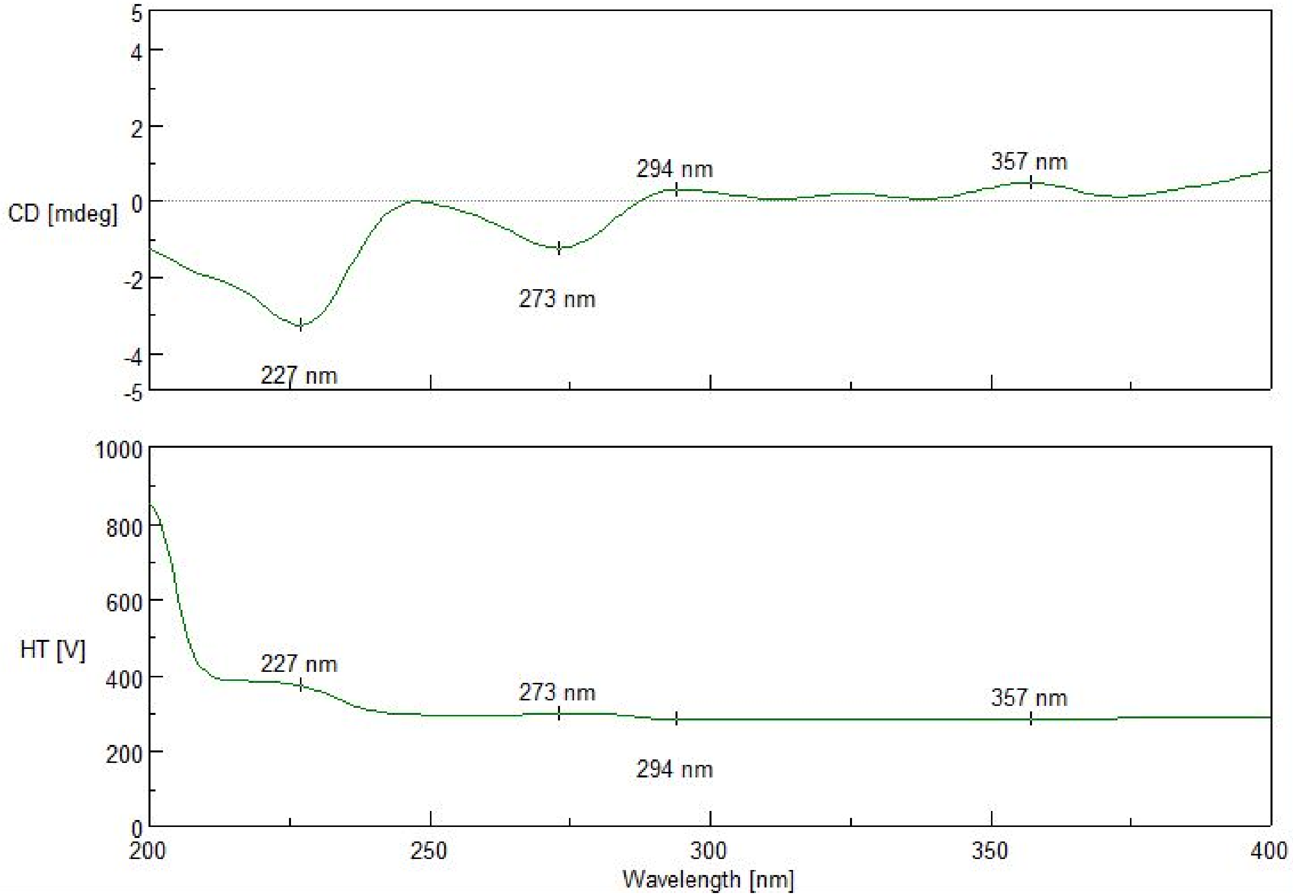

Figure S8. The ESIMS data of **2** (negative-ion mode)

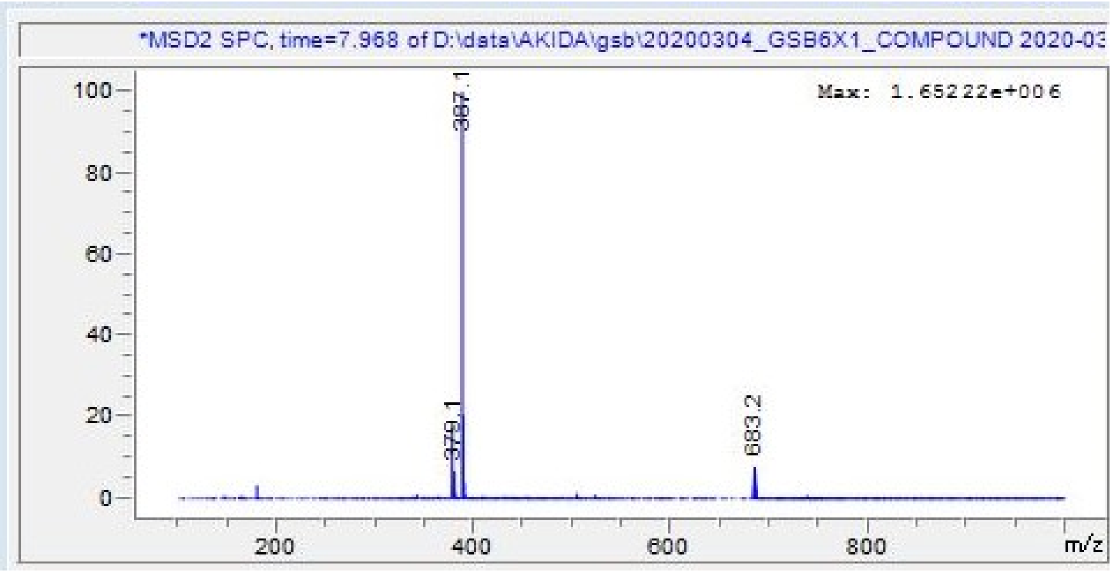

Figure S9. The ^{1}H NMR spectrum of **2** (CD_3OD, 850 MHz)

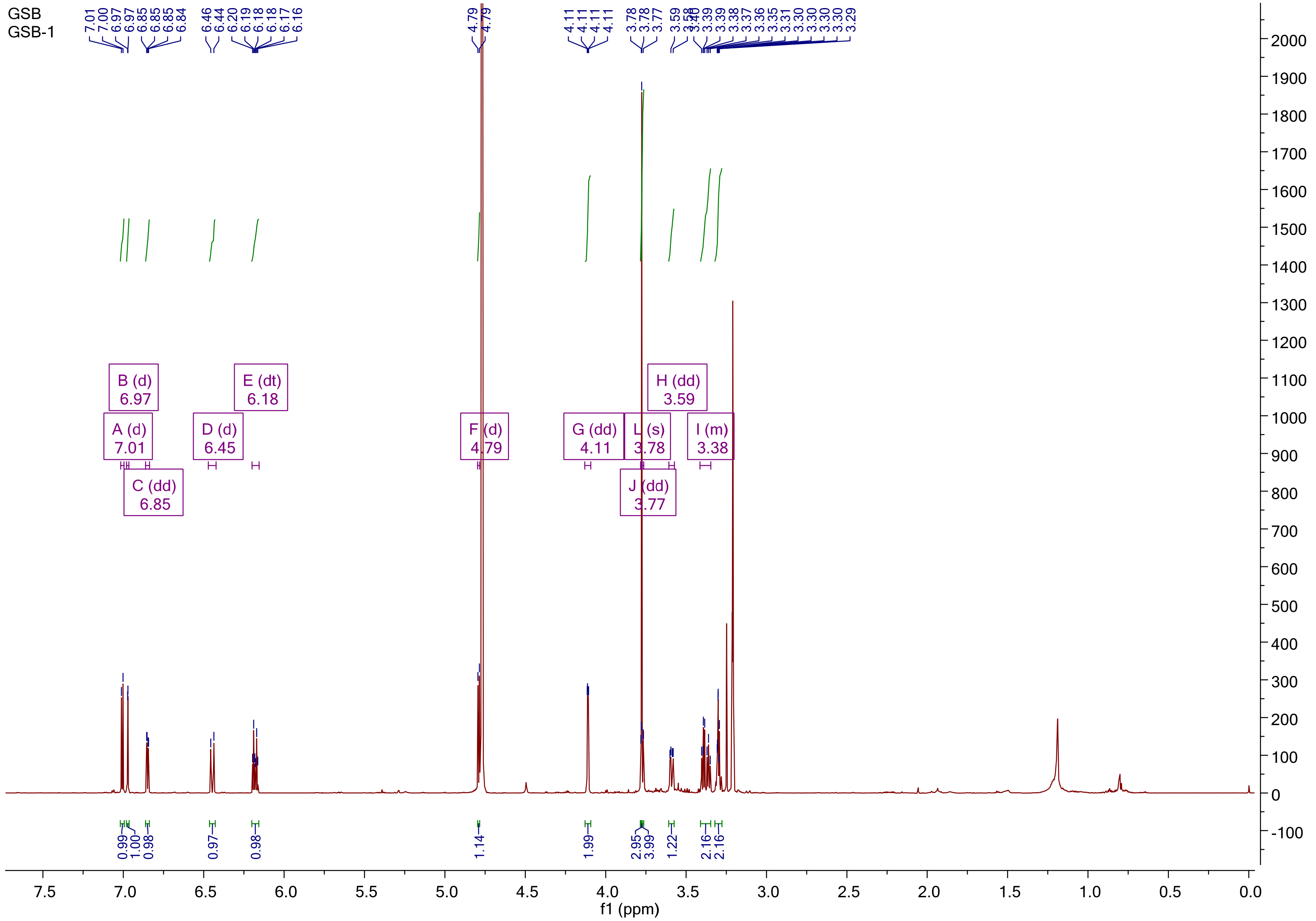

Figure S10. The ESIMS data of **3** (negative-ion mode)

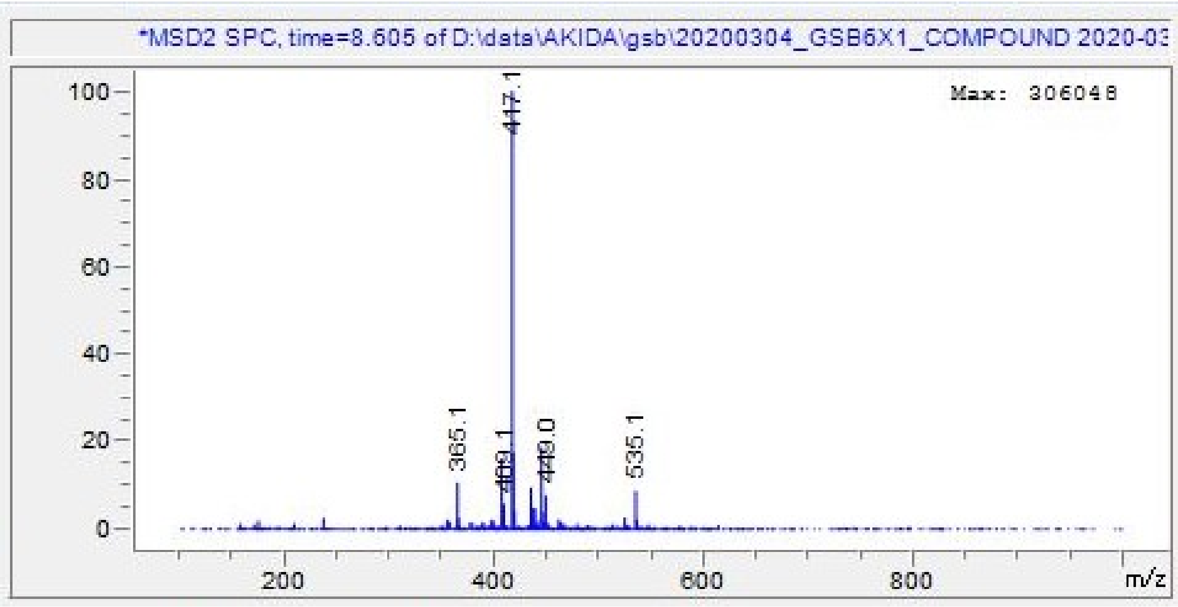

Figure S11. The ^{1}H NMR spectrum of **3** (CD_3OD, 850 MHz)

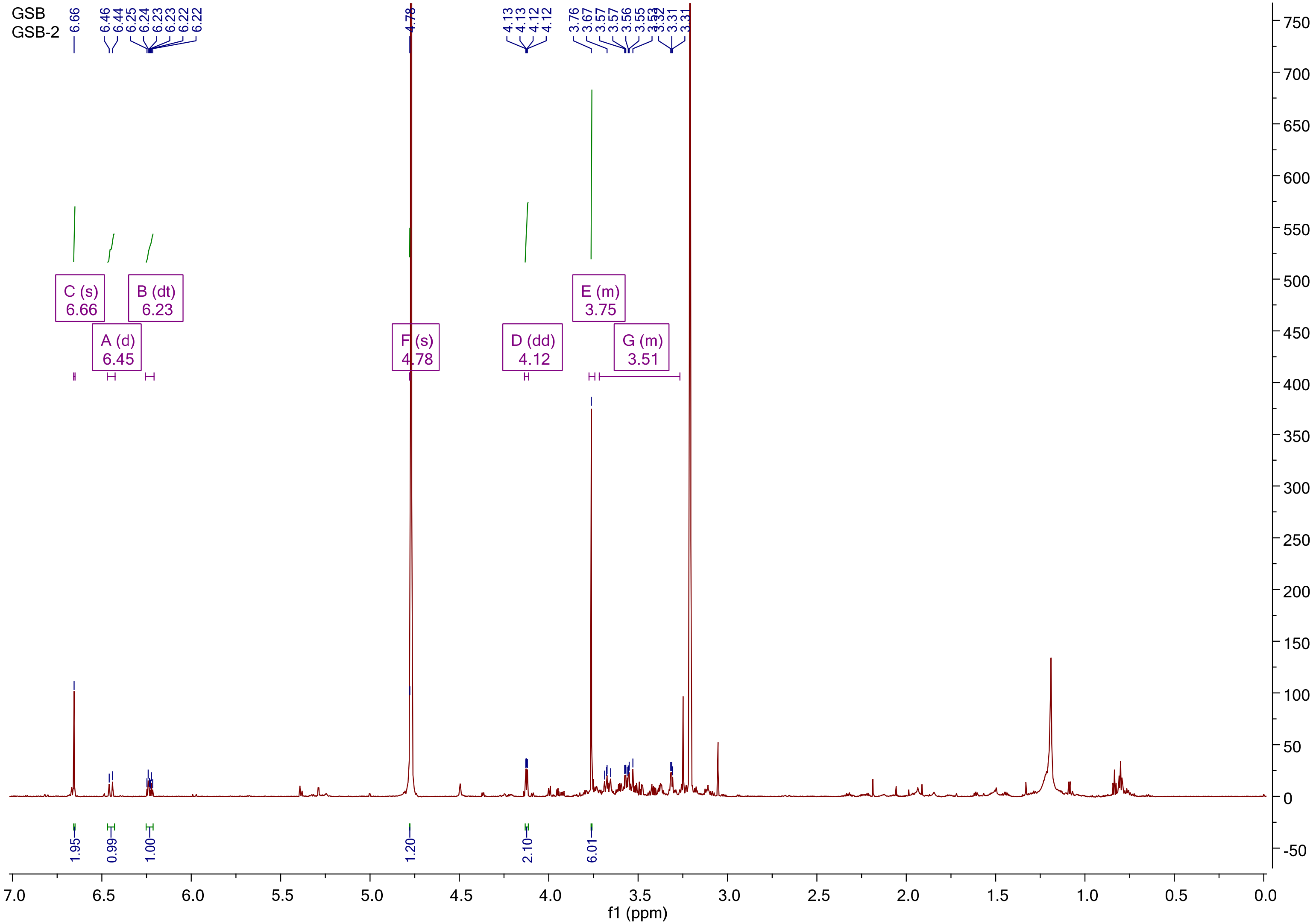

Figure S12. The ESIMS data of **4** (negative-ion mode)

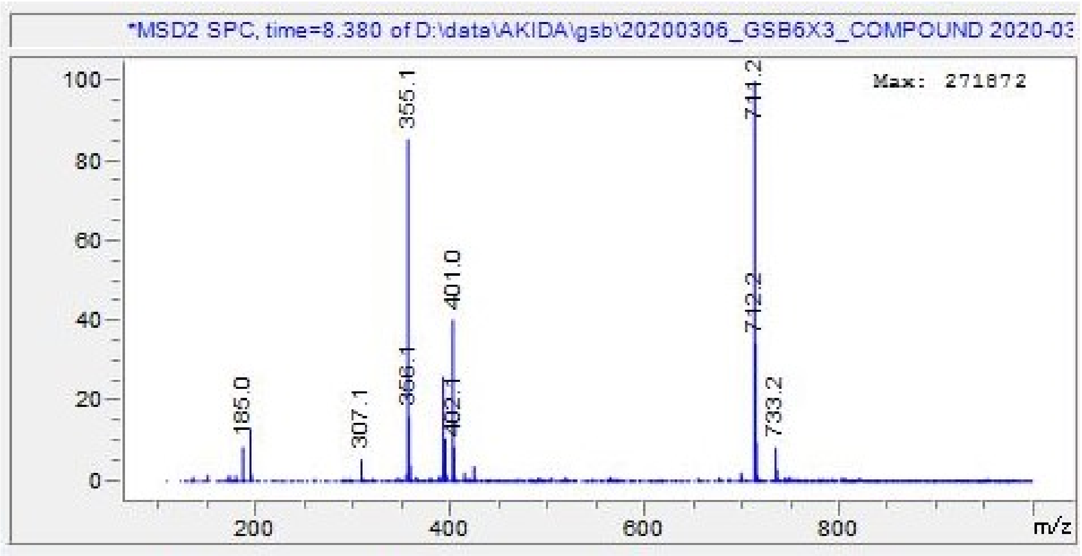

Figure S13. The ^{1}H NMR spectrum of **4** (CD_3OD, 850 MHz)

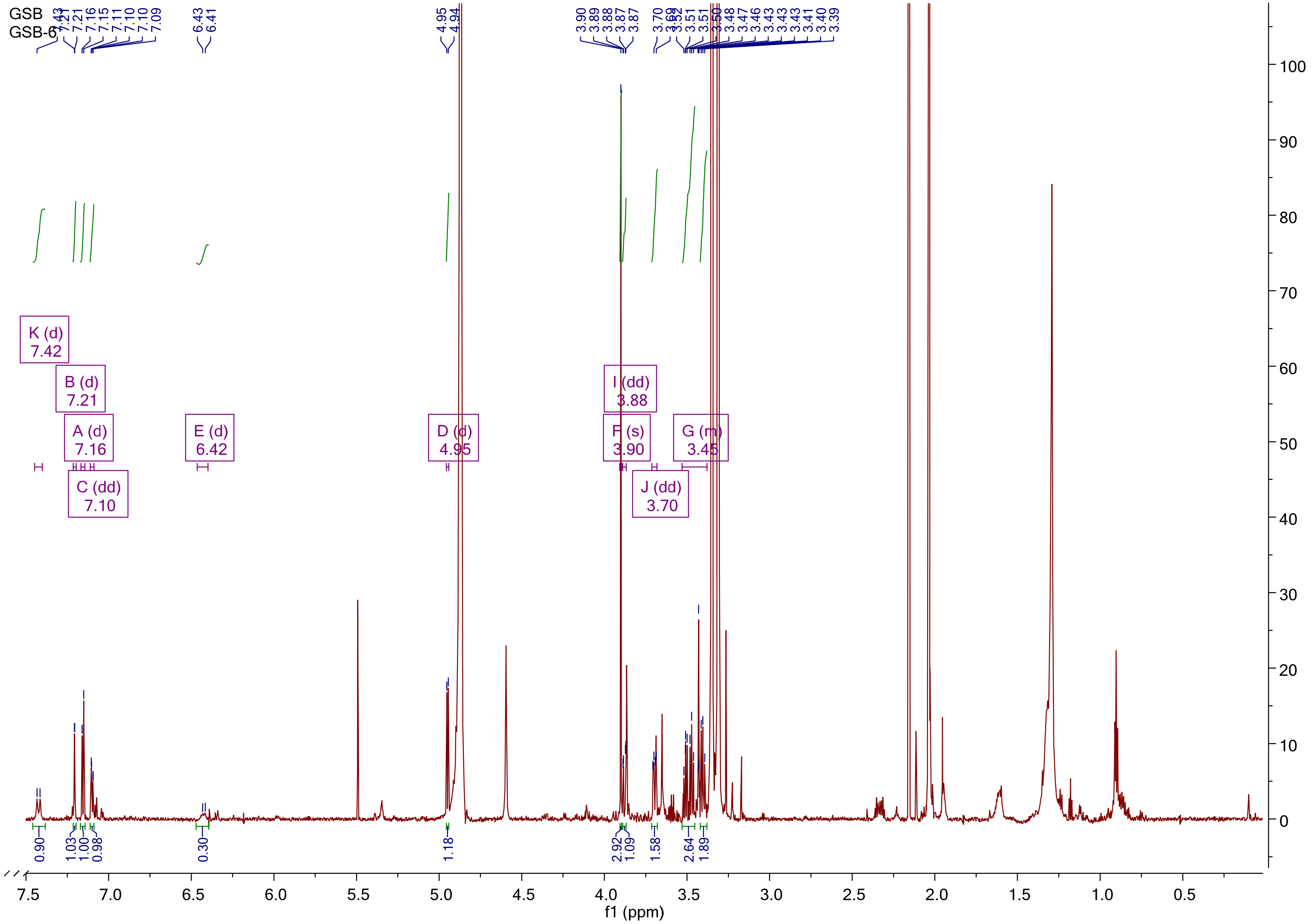

Figure S14. The ESIMS data of **5** (negative-ion mode)

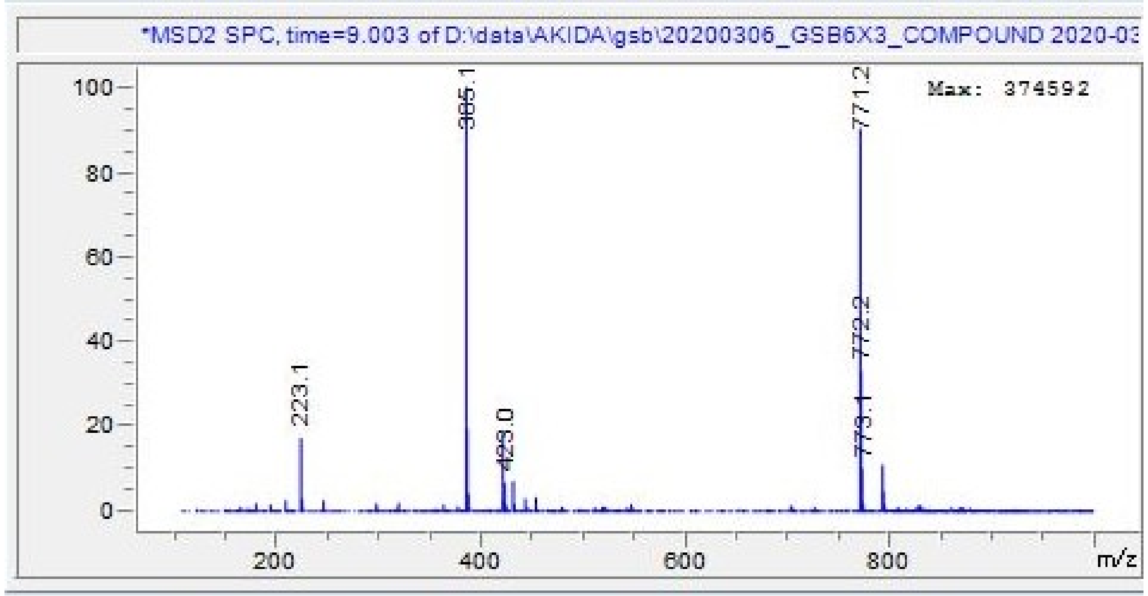

Figure S15. The ^{1}H NMR spectrum of **5** (CD_3OD, 850 MHz)

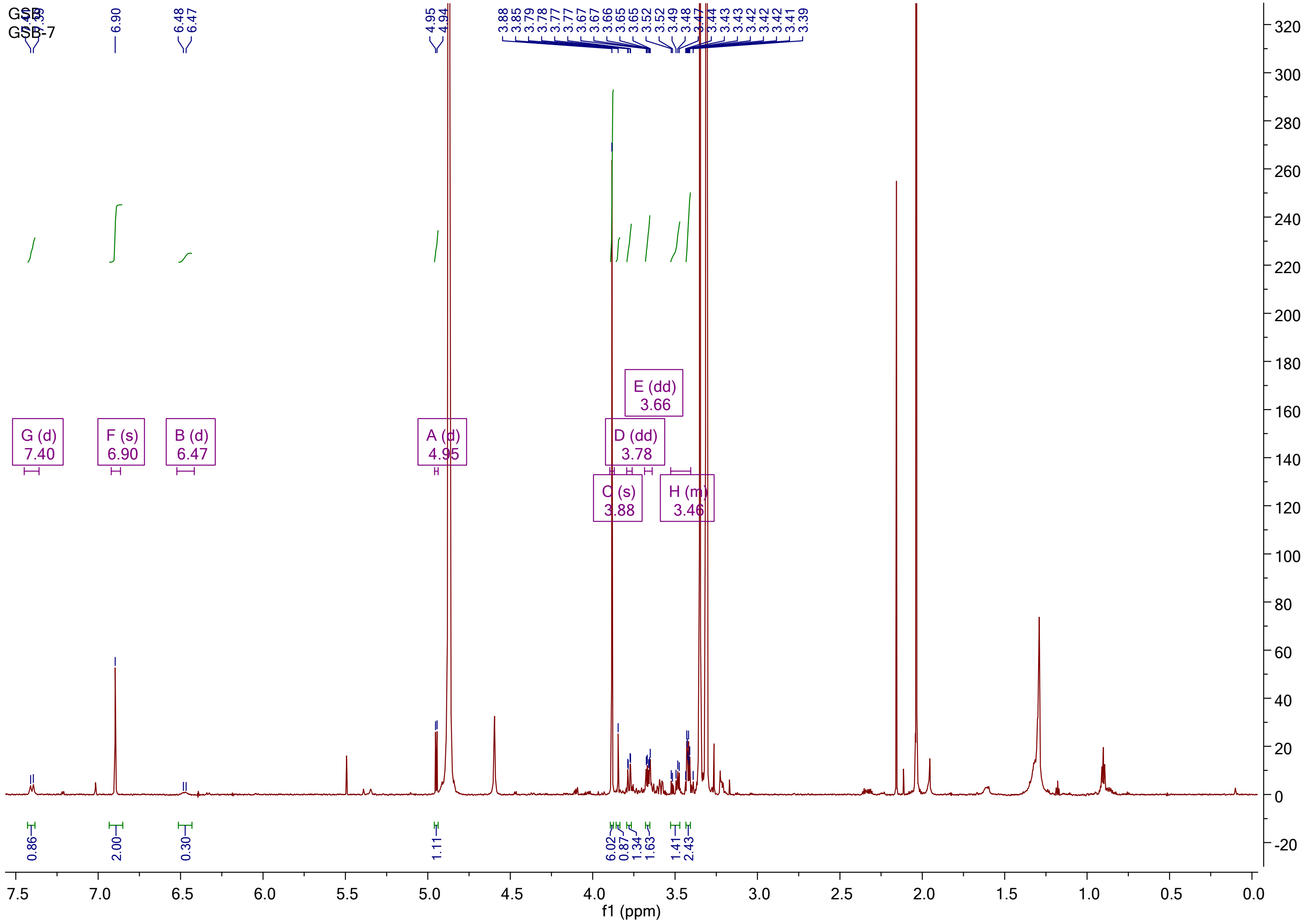

Figure S16. The ESIMS data of **6** (positive-ion mode)

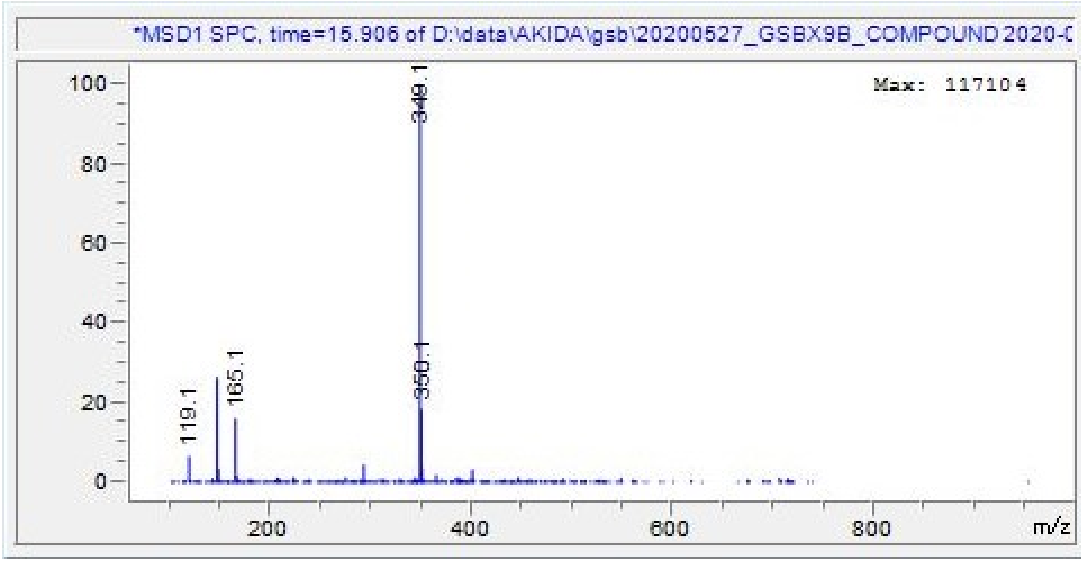

Figure S17. The ^{1}H NMR spectrum of **6** (CD_3OD, 850 MHz)

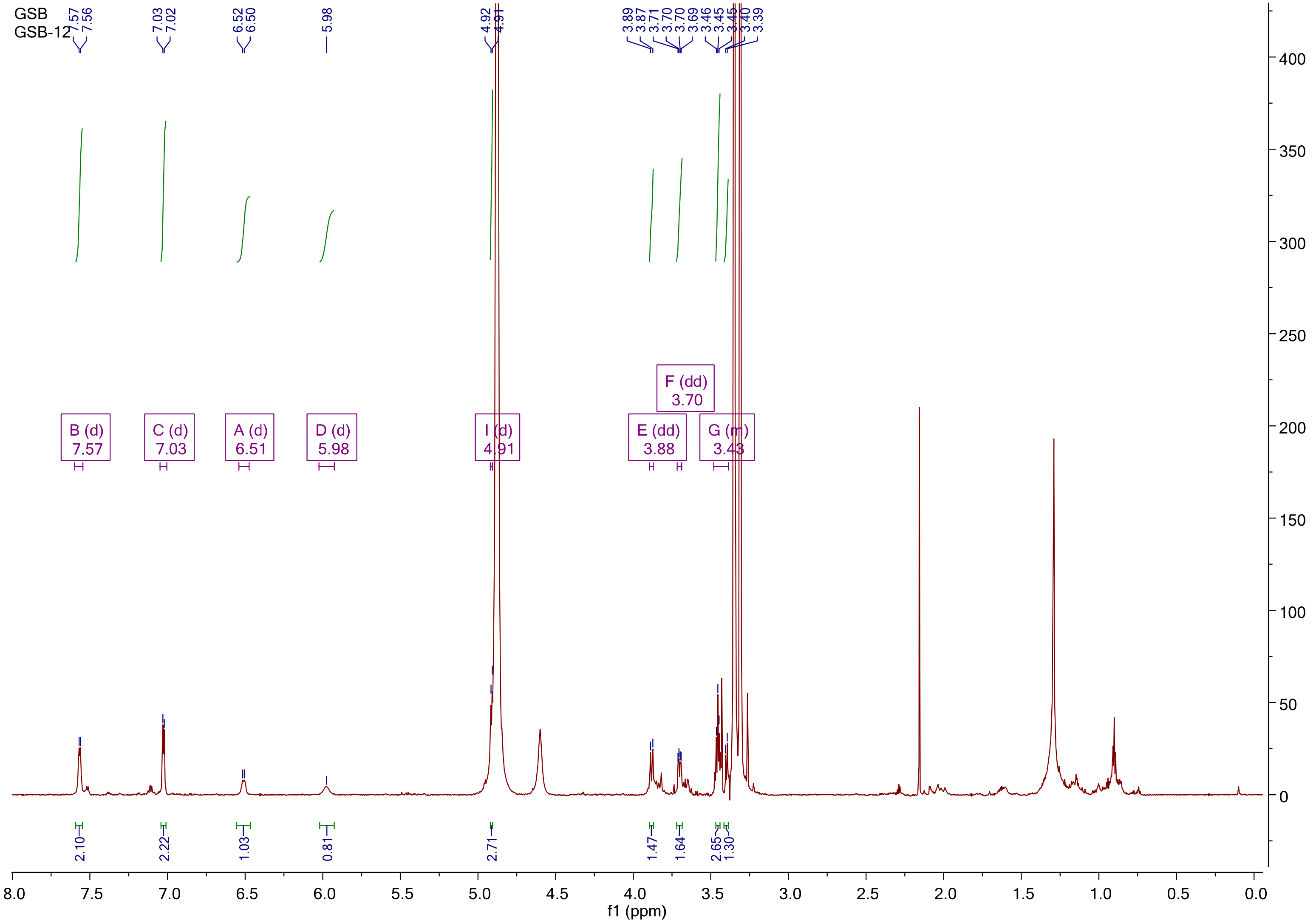

General experimental procedure

Optical rotations were measured using a Jasco P-2000 polarimeter (Jasco, Easton, MD, USA). Infrared (IR) spectra were recorded with a Bruker IFS-66/S FT-IR spectrometer (Bruker, Karlsruhe, Germany). Ultraviolet (UV) spectra were acquired on an Agilent 8453 UV-visible spectrophotometer (Agilent Technologies, Santa Clara, CA, USA). Circular dichroism spectra were measured on a Jasco J-1500 spectropolarimeter (Jasco). NMR spectra were recorded using a Bruker AVANCE III HD 850 NMR spectrometer with a 5 mm TCI CryoProbe, operated at 850 MHz (^{1}H) and 212.5 MHz (^{13}C). The chemical shifts were represented in ppm (δ) for ^{1}H and ^{13}C NMR analyses. Preparative and semi-preparative HPLC was performed using a Waters 1525 Binary HPLC pump with a Waters 996 photodiode array detector (Waters Corporation, Milford, MA, USA) using an Agilent Eclipse C18 column (250 × 21.2 mm, 5 µm; flow rate: 5 mL/min; Agilent Technologies) and a Phenomenex Luna Phenyl-hexyl 100 Å column (250 × 10 mm, 5 µm; flow rate: 2 mL/min; Phenomenex, Torrance, CA, USA). LC/MS analysis was performed on an Agilent 1200 Series HPLC system, equipped with a diode array detector and 6130 Series ESI mass spectrometer, and an analytical Kinetex C18 100 Å column (100 × 2.1 mm, 5 µm; flow rate: 0.3 mL/min; Phenomenex). All HRESIMS data were obtained using an Agilent G6545B quadrupole time-of-flight (Q-TOF) mass spectrometer (Agilent Technologies). Silica gel 60 (230-400 mesh; Merck, Darmstadt, Germany) was used for column chromatography. Diaion HP-20 (Mitsubishi Chemical, Tokyo, Japan) was used for open-column chromatography. Thin-layer chromatography was performed with precoated silica gel F_{254} plates and RP-C_{18} F_{254s} plates (Merck) and spots were detected under UV light or by heating, after spraying with anisaldehyde-sulfuric acid.

Plant material

The whole fruits of *G. biloba* were collected in the campus of Sungkyunkwan University, Suwon, Korea, in October 2019, and the plant was identified by one of the authors (K.H.K.). A voucher specimen of the material (GBF-2019-10) was deposited in the herbarium of the School of Pharmacy, Sungkyunkwan University, Suwon, Korea
